# Nuclear singlet multimers (NUSIMERs) with long-lived singlet states†
†Electronic supplementary information (ESI) available: Details on the synthesis of the NUSIMERs, additional spectra as well as a more detailed description on the singlet relaxation time measurements. See DOI: 10.1039/c8sc02831a


**DOI:** 10.1039/c8sc02831a

**Published:** 2018-10-25

**Authors:** Philip Saul, Salvatore Mamone, Stefan Glöggler

**Affiliations:** a NMR Signal Enhancement Group , Max-Planck-Institutefor Biophysical Chemistry , Am Faßberg 11 , 37077 Göttingen , Germany . Email: stefan.gloeggler@mpibpc.mpg.de; b Center for Biostructural Imaging of Neurodegeneration of UMG , Von-Siebold-Straße 3A , 37075 Göttingen , Germany

## Abstract

Magnetic resonance (NMR) is a powerful tool in chemical analysis, structure determination and in medical diagnostics. Developing novel biological sensors for this field holds promise to better investigate protein structures or target diseases more efficiently. Herein, we explore nuclear spin singlet states in dendritic macromolecules as a platform molecule to develop stimuli responsive probes. We have developed a nuclear singlet multimer (NUSIMER) based on a generation 5 poly(amidoamine) dendrimer (PAMAM) which contains on average about 90 accessible nuclear spin singlet states with lifetimes up to 10-fold longer than the *T*_1_ relaxation times (up to 10 seconds *T*_s_*vs. T*_1_ < 0.5 seconds) in a single molecule. We demonstrate little influence on the singlet lifetime in phosphate buffer (H_2_O) and a high viscosity gel environment in the presence of paramagnetic oxygen. Additionally, we demonstrate an increase in singlet lifetime upon the release of a protective chemical moiety from the NUSIMER following a stimulus, whereby no change in longitudinal relaxation time is observed. The robustness and change in singlet lifetime of the NUSIMER holds promise for the development of a novel type of biosensors.

## Abstract 2

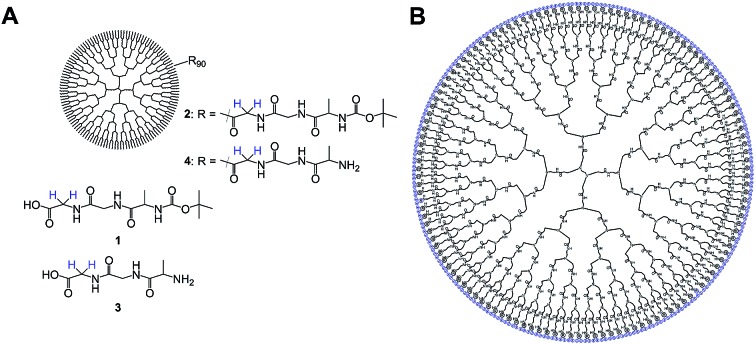
We are introducing nuclear spin singlet multimers which are molecules that contain several nuclear singlet states that can be populated at the same time.

## Introduction

Nuclear spin singlet states are spin states that are not directly observable in NMR. However, observable magnetization can be transformed and stored in a singlet state and retrieved back at a later time.^1^–^31^ In a singlet state, two given *l* = 1/2 nuclei are brought into a state in which they couple into an effective spin 0 state. Spins in such a state are immune to intramolecular dipole–dipole relaxation, which is one of the main nuclear spin relaxation mechanisms.^31^ This can lead to long equilibration times between singlet and triplet state which we refer to as singlet lifetimes *T*_s_. They can be significantly longer than the corresponding spin-lattice relaxation times *T*_1_. Long *T*_s_ can be extremely useful in hyperpolarized NMR, in which nuclear spin polarization is dramatically enhanced and more than four orders of magnitude in signal-to-noise ratio can be gained.^32^ Singlet states were used to observe slow processes on timescales longer than it was normally accessible by standard NMR methods, including the diffusion processes of certain compounds in solution or changes in the close surroundings of the observed compound.^6^,^10^,^16^ Although the singlet lifetime depends on the rotational correlation time of the molecule under study (and therefore small molecules are typically investigated), it was shown that singlet states between proton pairs can be populated in proteins.^7^,^33^ This is probably due to the local degree of freedom in motion. First steps have been made to develop nuclear spin singlet states into biological probes.^16^,^34^,^35^ In order to develop a bio-probe, sensitivity needs to be high enough that the singlet states can be observed at the location of interest in an organism. Hyperpolarization may be used to boost the NMR signal, and so far minute long singlet lifetimes were observed in organic solvent or in the absence of oxygen *in vitro*.^8^,^13^,^23^,^25^ Observing protein binding has so far been the main biological-oriented application of singlets but due to the short *T*_s_ of protons and fluorine nuclei used in these experiments, long traceability *in vivo* may not be possible.^16^,^17^,^29^ In order to circumvent sensitivity issues we have designed macromolecules based on a dendrimer in which an average of 90 pairs of nearly equivalent protons per molecule are present. Our specific goal here is not to hyperpolarize the compound but rather rely on thermal polarization and potentially increase the detections sensitivity by averaging. Hyperpolarization may however become an option in the future upon the discovery of *e.g.* molecules with hour-long singlet states that can be attached to the here proposed structures. The proton pairs in the dendrimers can be simultaneously toggled between magnetization and singlet states, allowing for the detection of a double digit μM concentration of the macromolecules in a two-scan experiment. Proton pairs were chosen since they have a proven versatility and a high gyromagnetic ratio for increased NMR sensitivity.^36^ The robustness of the proton singlet state was investigated by exposing them to paramagnetic oxygen in air, non-deuterated solvents/buffers and to a high viscosity environment based on an agarose–phosphate buffer gel. Additionally, we have investigated the possibility to detect the effect of external stimuli on the NUSIMER. A NUSIMER with acid labile moieties significantly changes its nuclear singlet lifetime upon a stimulus that leads to cleavage of the moieties while the corresponding longitudinal relaxation times remain unaffected.

### General idea

The aforementioned effects have been shown by investigating the singlet state decay in proton pairs in the CH_2_ groups of a glycine-glycine-alanine (GGA) tripeptide sequence, in the free peptide form as well as when attached to a dendrimer. The GGA sequence has been chosen since the two C_α_ bonded protons in the terminal glycine form a strongly coupled proton pair that appears as a single line in the ^1^H NMR spectrum, which increases the signal-to-noise-ratio.^27^ The spin lattice relaxation is mainly governed by intramolecular dipole–dipole interactions due to the close proximity of the two protons in glycine.^37^ The chemical shift anisotropy, introduces a field dependent relaxation mechanism,^13^ which is negligible in the magnetic field used for the experiments *B*_0_ = 7.05 T. In order to be able to bring the magnetization into a singlet state, there needs to be a slight difference in the magnetic environment surrounding the two observed protons.^31^ By using the tripeptide which contains an alanine that adds a stereogenic center to the molecule remote to the two observed nuclei, we create the slight difference in chemical shift needed to populate a singlet state, while keeping a strongly coupled system.^37^ Furthermore, in strongly coupled systems, singlet states can be maintained without decoupling,^31^ thus increasing the scope for biological applications. Decoupling may lead to heating of the sample, which poses a problem especially for *in vivo* experiments.

Dendrimers are highly symmetric polymeric structures built up from a symmetric core with branches that in turn split up several times to generate a ball like structure with a high number of terminal functional groups.^38^ The generated structure allows for derivatization on terminal groups leading to a high density of various possible desired terminal modifications. This possibility has gained some interest especially in medical applications ranging from drug delivery^39^,^40^ and cytotoxica^41^–^43^ to fluorescence imaging.^44^ Apart from the aforementioned applications, possible modifications with gadolinium have been reported.^45^,^46^ In these cases the goal was to make use of the fact that dendritic structures can hold multiple gadolinium-ions on the terminal groups, to enhance *T*_1_ of surrounding water and thus being able to generate a contrast in MRI.

## Results and discussion

### Modification of the dendrimer

**Fig. 1:**
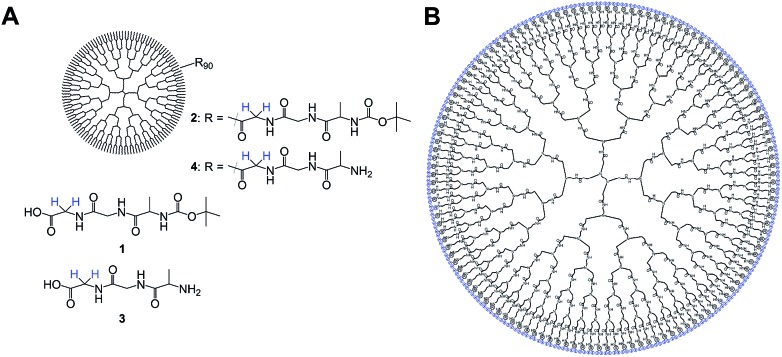
(A) Modifying G5-PAMAM dendrimers by adding a glycine-glycine-alanine sequence with (**1**) and without Boc protection group (**3**) leads to the structures **2** and **4**. (B) The resulting structure bears up to 128 tripeptides thus generating up to 128 accessible singlet states (blue) in the same molecule.

In this work we modified a generation 5 poly(amidoamine) (G5-PAMAM) dendrimer by adding terminal tripeptides, namely the previously discussed glycine-glycine-alanine sequence **3** as well as the same tripeptide additionally containing a large *tert*-butoxycarbonyl (Boc) group cleavable in acidic environment **1** (Fig. 1).

The obtained compounds G5-PAMAM-GGA-N-Boc **2** and G5-PAMAM-GGA-NH_2_**4** can bear up to 128 tripeptide domains per molecule in which singlet states are accessible and we found an average surface coverage of about 70%, *i.e.* 90 peptides, as shown in the ESI.†

### NMR measurements

**Fig. 2:**
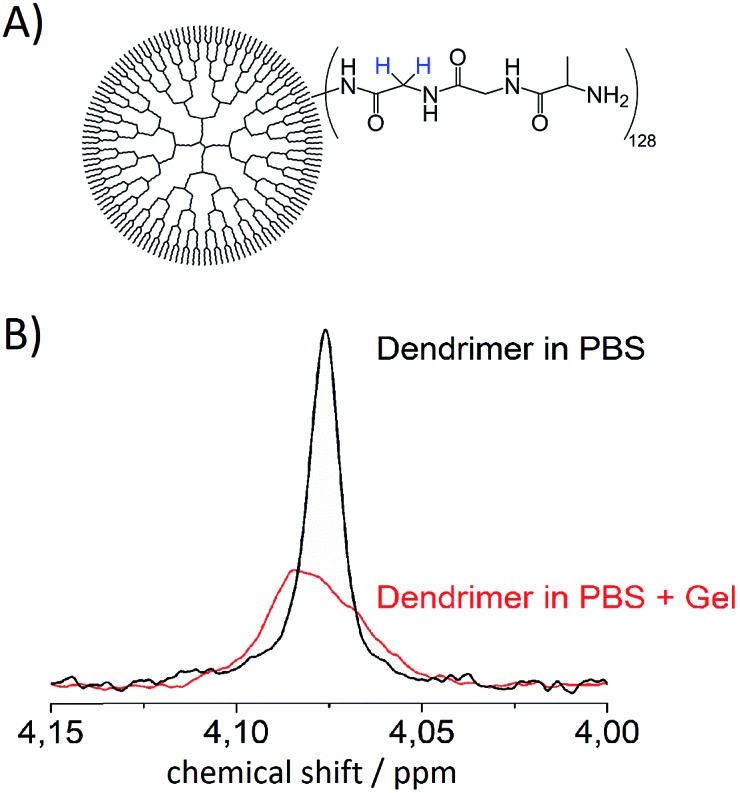
(A) Schematic representation of **4**. (B) Comparison of the NMR signal obtained from magnetization previously stored in a singlet state of the protons in (A) (blue) in aqueous PBS solution (black) and the signal in aqueous PBS agarose gel (red). The spectrum shown arises from a 100 μM solution of **4**.

Since the system is strongly coupled with an undetectable chemical shift difference, the SLIC sequence^47^ has been used to access the singlet state. A two-step phase cycling was used to cancel the signal that has not passed through the singlet state.^48^ This singlet state sequence acts as a filter, allowing for samples being measured in aqueous phosphate buffered saline (PBS, pH = 7.4) solutions while suppressing water as well as other signals in the spectrum, see Fig. 2.

Additional measurements in an agarose gel (0.6 wt%) prepared with aqueous PBS have been performed. This environment is used to simulate the viscosity of the human brain tissue.^49^ The linewidth of the singlet signal in the agarose gel sample is broader (18 Hz) and the signal intensity is a little lower than in the PBS solutions (Fig. 2). Both in PBS and in the agarose/PBS gel, the *T*_s_ times are longer than *T*_1_. The relaxation times in agarose are comparable to those measured in PBS solution, which is true for the NUSIMERs as well as for the free tripeptides (Table 1). The measured relaxation times for the free tripeptides at 37 °C are longer than the ones of the respective NUSIMERs. In D_2_O solution, the singlet lifetime for the free tripeptide **1** was *T*_s_ = 13 s whereas **2** showed a *T*_s_ = 10.2 s. The *T*_1_ values are significantly shorter, *T*_1_ = 0.90 s for 1 and *T*_1_ = 0.43 s for **2**, respectively. The *T*_s_ in the two different NUSIMERs are influenced quite significantly by the presence of the Boc protection group. The removal of this group decreases the singlet life-time from *T*_s_ = 10.2 s for **2** to *T*_s_ = 6.9 s for **4** whereas the *T*_1_ values are not influenced, see Table 1. Such behavior is accentuated in PBS solution. The singlet state in the NUSIMER containing the Boc protection group **2** is not accessible anymore in aqueous PBS. This accessibility tuning by ions is not observed in compound **4**. Upon cleavage of the Boc group in non-degassed PBS solution, the singlet state becomes accessible again and a *T*_s_ = 1.81 s was obtained whereas the longitudinal relaxation changes within 20% (*T*_1_ = 0.45 s and 0.35 s). The influence of the agarose gel environment on *T*_1_ and *T*_s_ is within 20% as well (Table 1). Furthermore we observed that *T*_s_ measurement without decoupling in **4** did not change and was *T*_s_ = 6.89 s. This has been shown to not be the case in weakly coupled systems.^31^

**Table 1:** Singlet state lifetimes *T*_s_ and relaxation times *T*_1_ of different samples in different solvents. Solvents have not been degassed prior to measurements

Compound^*a*^	Solvent	*T*_1_ [s]^*b*^	*T*_s_ [s]^*c*^
**1**	D_2_O	0.90 ± 0.04	13 ± 2
**1**	H_2_O (PBS)	0.70 ± 0.07	3.87 ± 0.06
**1**	Agarose (PBS)	0.6 ± 0.1	3.1 ± 0.2
**2**	D_2_O	0.43 ± 0.01	10.2 ± 0.9
**2**	H_2_O (PBS)	0.45 ± 0.01	n.a.
**2**	Agarose (PBS)	0.38 ± 0.26	n.a.
**3**	D_2_O	1.60 ± 0.02	30 ± 3
**3**	H_2_O (PBS)	1.54 ± 0.17	7.8 ± 0.6
**3**	Agarose (PBS)	1.14 ± 0.09	8.59 ± 0.06
**4**	D_2_O	0.49 ± 0.01	6.9 ± 0.6
**4**	D_2_O	0.49 ± 0.01	6.9 ± 0.5^*d*^
**4**	H_2_O (PBS)	0.35 ± 0.01	1.8 ± 0.2
**4**	Agarose (PBS)	0.43 ± 0.08	1.5 ± 0.1

^*a*^Concentrations of the free tripeptides are 12.8 mM. Concentrations for the NUSIMERs are 100 μM.

^*b*^ *T*_1_ relaxation times have been determined using the inversion recovery experiment. Two samples have been measured for each compound and the average is represented here.

^*c*^ *T*_s_ have been determined using the SLIC experiment^47^ with additional phase cycling.^48^ The average over two samples is represented in the table.

^*d*^Measured values without applying a spin-locking field.

### A stimulus responsive probe

**Fig. 3:**
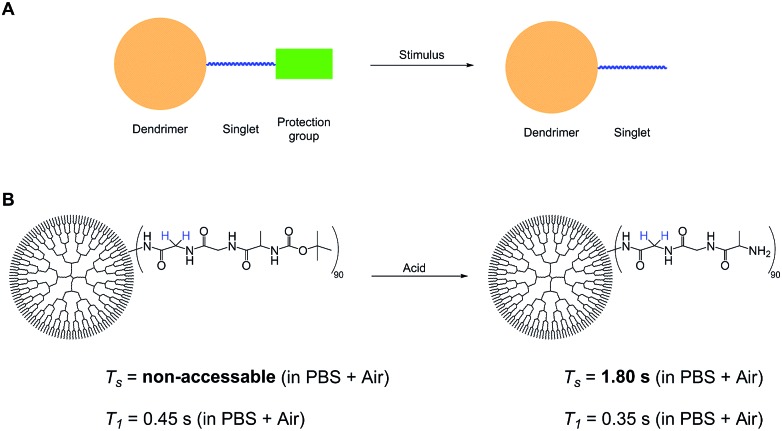
(A) Removal of the Protection group with a stimulus frees the singlet state containing chain thus making the “hidden” singlet state accessible. (B) Treatment of **2** with acid removes the protection group to form **4**. The singlet state becomes accessible while the *T*_1_ changes within 20%.

These observations lead us to propose a stimulus responsive bio-probe based on the design and experiments shown in Fig. 3. Here a protection group is cleaved in presence of a stimulus, triggering the NUSIMER accessibility and modifying singlet lifetimes. In the herein investigated case, detection of an acidic environment is possible by *T*_s_ time measurements. Other possible stimuli include interaction with certain proteins as well as ion detection by accessibility of certain singlet states. Further investigations are currently conducted in our group. With a view on potential translatability in the future, MRI experiments in mouse models have been performed by an intravenous injection of 4 mg of PAMAM to acquire good images.^50^ Toxicity studies have shown that in particular hydroxyl terminated dendrimers are non-toxic in mice even in doses over 400 mg kg^–1^.^51^ This amount of substrate is much higher than investigated here. Although *in vivo* singlet-MRI is still a long path to go, our presented dendrimers promise a large step towards this direction.

## Conclusions

In summary, we have shown that by adding a glycine-glycine-alanine sequence on the terminal groups of a G5-PAMAM dendrimer, conditions are met that allow for populating on average 90 long lived nuclear spin singlet states per molecule at the same time. The NUSIMER long-lived states remain stable in non-degassed PBS environment as well as in an agarose gel used to simulate brain tissue, suggesting potential applications in biomedical imaging. The significant change in specifically singlet lifetimes upon the cleavage of the Boc protection group makes NUSIMERs highly interesting as possible stimuli-responsive biosensors. This effect cannot be observed in the longitudinal relaxation. The chemical shift proximity of the proton pair signal makes decoupling for singlet maintenance unnecessary. These properties, along with detectability in μM concentrations, make the NUSIMERs possible candidates for biological applications. Here, we have demonstrated the concept of NUSIMER in a group of highly versatile macromolecular compounds with conceivable biological application in the future.

## Conflicts of interest

There are no conflicts to declare.

## Supplementary Material

Supplementary information
