# Supplementary material for: Nuclear singlet multimers (NUSIMERs) with long-lived singlet states
Source: Chem Sci. 2018 Oct 25;10(2):413–7. doi: 10.1039/c8sc02831a (PMC6334717; doi:10.1039/c8sc02831a)
Supplement: Supplementary file 1 [file SC-010-C8SC02831A-s001.pdf]

Electronic Supplementary Material (ESI) for Chemical Science.
This journal is © The Royal Society of Chemistry 2018

Supporting Information

Nuclear singlet multimers (NUSIMERs) with long-lived singlet states

Philip Saul[a,b], Salvatore Mamone[a,b] and Stefan Glöggler*[a,b]

Chemicals

The Boc protected tripeptide **1** was purchased from TCI chemicals and used as received. Generation 5 poly(amidoamid) dendrimer (G5-PAMAM) was purchased from SigmaAldrich as a methanol solution (5wt.%). The methanol was removed prior to the synthesis. Gibco™ phosphate buffered solution (pH=7.4 ,10x) was purchased from ThermoFisher Scientific.

Syntheses of (2) and (4)

*Synthesis of G5-PAMAM-GGA-NH-Boc **(2)***

For the synthesis of **2** the following synthesis was conducted: G5-PAMAM was dissolved in a mixture of dimethylsulfoxide (DMSO) and dimethyl formamide (DMF) (4:1) and 256 equivalents of **1** was added with 256 equivalents of di-isopropyl-ethyl-amine (DIPEA) and 256 equivalents of *N*-(3-diaminopropyl)-*N'*-ethylcarbodiimide hydrochloride (EDC). The resulting solution was stirred at room temperature for 24 h and the solvent was removed. The obtained solid was taken up in H_2O and dialyzed against H_2O for one week. Afterwards the solvent was removed to obtain a colorless solid.

*Synthesis of G5-PAMAM-GGA-NH_2 **(4)***

2 was dissolved in a mixture of Dichloromethane (DCM) and Trifluoroacetic acid (TFA) (1:1) for 30 minutes. Afterwards the solvent was removed and the obtained crude product was dissolved in H_2O and dialyzed against H_2O for one week. Afterwards the solvent was removed to obtain an amber highly viscous liquid.

NMR experiments

NMR experiments have been performed on a BRUKER AVIII 300 MHz spectrometer. The parameters for the respective experiments are shown in the following figures.

Estimation of the mean number of tripeptides per dendrimer unit

The mean derivatization number has been estimated by using pyrazine as an internal standard in quantitative NMR experiments. A solution of **2** and **4** has been measured with an internal standard of

50 mM pyrazine. The amount of **2** and **4** in the solutions was calculated to be 100 µM assuming complete derivatization. Thus, the assumed molarity of tripeptide residues would be 12.8 mM. The actual molarity of tripeptide residues has been calculated by comparing the integral of the pyrazine signal with the integral of the methyl Protons of the Alanine. The actual derivatization is 0.69 for **2** and of 0.68 for **4**. This results in a mean coverage of about 90 tripeptides per NUSIMER.

On the accessibility of the singlet state in (2)

As mentioned in the main article the singlet state in the signal at 3.87 ppm was not accessible any more upon PBS addition. As shown in figure S1, measurements performed on a BRUKER 400 MHz machine in D_2O showed a difference in chemical shift in the signal at 3.80 ppm which was not visible in PBS solution anymore.

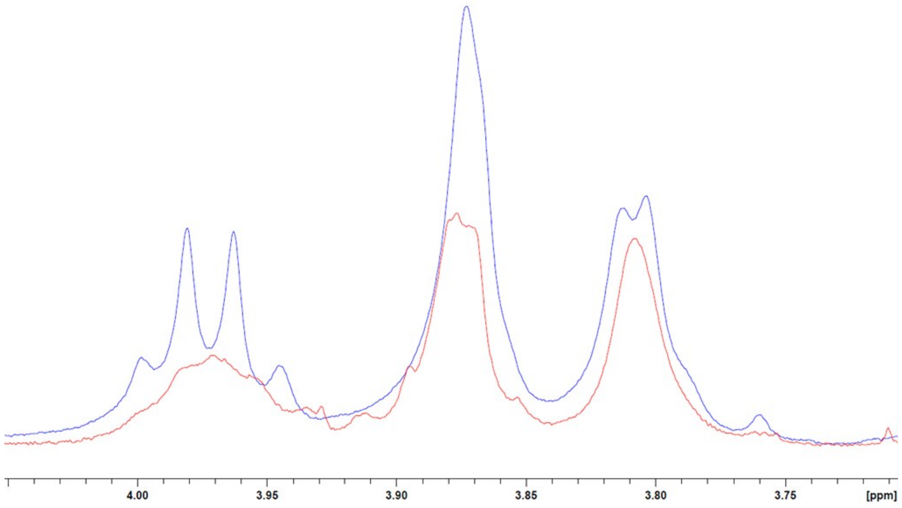

Figure S1:Expansion of the ^{1}H NMR spectrum of **2** showing from left to right the signal of the CH proton of alanine (quartet centered at 3.97ppm) and of the CH_2 protons of the two glycine residue at 3.87 ppm and 3.81 ppm, respectively . In the spectrum measured in D_2O (blue) a chemical shift difference is visible, whereas in PBS solution (red) the chemical shift difference can not be resolved anymore.

Overall, this region of the NMR spectrum seems to be highly influenced by the presence of PBS. In the same spectrum the signals arising from the methyl group of the alanine, as well as the signals of the Boc protection group, show a change in the other direction, showing higher resolution and the appearance of a splitting of the signals (figure S2).

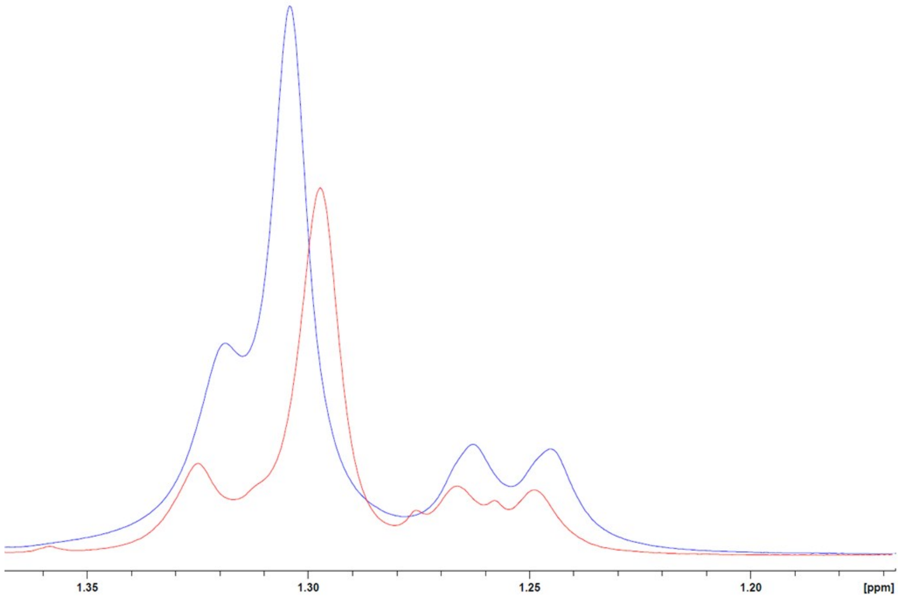

Figure S2: Signals of the Boc protection group and the methyl group of the alanine in D_2O (blue) and in PBS solution (red). A splitting of the signal in the alanine (1.26 ppm) is clearly visible upon addition of PBS.

This observation leads us to assume a strong interaction of the NUSIMERs with the ions in the PBS solution. Further investigation of this phenomenon will be subject of our future research. Here, we speculate that in presence of PBS, the interaction leading to a chemical shift difference in the glycine signal at 3.81 ppm is influencing the signal at 3.87 ppm in the same way, making the singlet state inaccessible.

Singlet life-times have been investigated in D_2O for the signal at 3.81 ppm and a singlet relaxation time of T_s=2.51 ± 0.04 s. Removing the spin lock caused T_S to decrease significantly to T_s=0.73 ± 0.02 s. This reflects the observed change in chemical shift difference. For comparison T_S have been determined in H_2O (5 % D_2O) solutions without the addition of PBS for **2** as well. The singlet state in both signals has been accessible with T_s=1.08 s and T_s=3.46 s in the signals at 3.95 ppm and 4.02 ppm respectively, showing that the observed change in accessibility can be attributed to the PBS and is not dependent on the deuteration level of the solvent.

Estimation of the recovered magnetization

The magnetization recovered after the end of the experiments have been estimated. For each of the signals the SLIC[1] pulse lengths and powers have been optimized and the singlet evolution time was set to 200 ms. For the protons of the glycine further away from the alanine residue we found that 5% of magnetization could be recovered (SLIC pulse length 310 ms, SLIC pulse power $6.6e^{-6}$ W). For the glycine protons closer to the alanine residue, 41% of the initial magnetization could be recovered (SLIC pulse length 50 ms, SLIC pulse power $8e^{-6}$ W). The theoretical maximum with the SLIC technique is 66% percent and our experiments show that optimization of the chemical shift difference between the two protons of interest can enhance the obtainable signal in the future.

Measurements of T_1

For the measurement of the T_1 relaxation times, an inversion recovery experiment has been conducted. The obtained plots are listed below. The values given in the main article are the mean values taken from the values obtained from two different samples.

Data	
t	Integrals
0.050	−0.791
0.075	−0.742
0.100	−0.699
0.150	−0.608
0.200	−0.523
0.250	−0.443
0.300	−0.368
0.350	−0.296
0.400	−0.226
0.500	−0.102
0.700	0.112
1.000	0.358
1.500	0.626
2.000	0.786
3.000	0.932
5.000	1.000

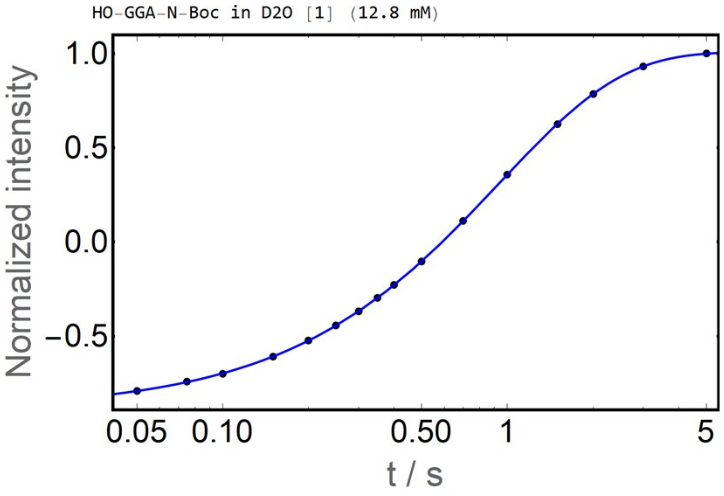

— $y=M1\times[1-\mathrm{Exp}(-t/\tau1)]+c1$

	Estimate	Standard Error	t-Statistic	P-Value
M1	1.89815	0.000722889	2625.79	1.33931×10^{-38}
τ1	0.933287	0.00105327	886.089	1.81974×10^{-32}
c1	−0.889667	0.000526786	−1688.86	4.15467×10^{-36}

Figure S3: Plot from the inversion recovery measurement of compound **1** in D_2O including the delays with the respective normalized integrals and the obtained T1 value.

Data	
t	Integrals
0.050	−0.839
0.075	−0.782
0.100	−0.729
0.150	−0.630
0.200	−0.537
0.250	−0.446
0.300	−0.363
0.350	−0.287
0.400	−0.213
0.500	−0.078
0.700	0.143
1.000	0.394
1.500	0.661
2.000	0.812
3.000	0.945
5.000	1.000

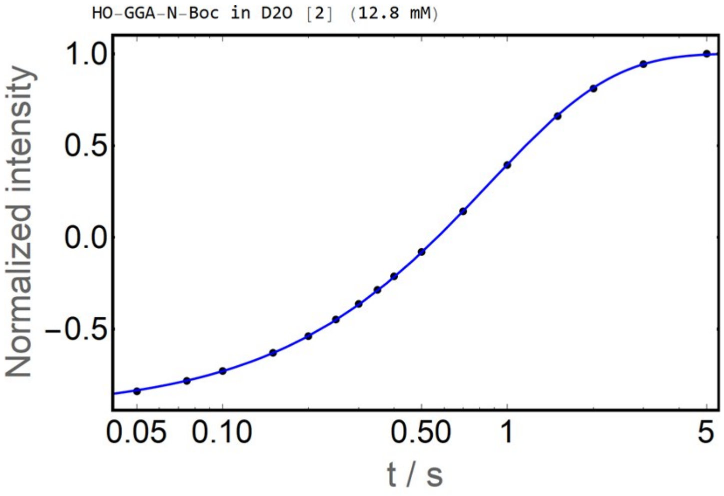

— $y=M1\times[1-\mathrm{Exp}(-t/\tau1)]+c1$

	Estimate	Standard Error	t-Statistic	P-Value
M1	1.94536	0.00283142	687.062	4.96859×10^{-31}
τ1	0.8541	0.00366579	232.992	6.3268×10^{-25}
c1	−0.943454	0.00218055	−432.668	2.02796×10^{-28}

Figure S4: Plot from the inversion recovery measurement of compound **1** in D_2O including the delays with the respective normalized integrals and the obtained T1 value.

Data	
t	Integrals
0.050	−0.574
0.075	−0.504
0.100	−0.486
0.150	−0.330
0.200	−0.248
0.250	−0.109
0.300	−0.019
0.350	−0.021
0.400	−0.049
0.500	0.178
0.700	0.271
1.000	0.581
1.500	0.805
2.000	1.000
3.000	0.700
5.000	0.896

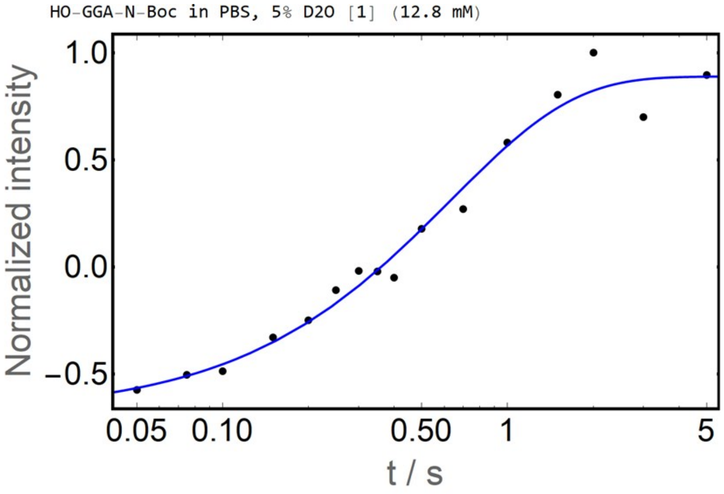

— $y = M1 \times [1 - \mathrm{Exp}(-t/\tau 1)] + c1$

	Estimate	Standard Error	t-Statistic	P-Value
M1	1.57426	0.068231	23.0724	6.21539×10^{-12}
$\tau 1$	0.630279	0.0756185	8.33499	1.4231×10^{-6}
c1	−0.685073	0.0599907	−11.4197	3.77838×10^{-8}

Figure S5: Plot from the inversion recovery measurement of compound **1** in PBS including the delays with the respective normalized integrals and the obtained T1 value.

Data	
t	Integrals
0.050	0.599
0.075	0.433
0.100	0.442
0.150	0.375
0.200	0.256
0.250	0.113
0.300	0.126
0.350	0.124
0.400	0.014
0.500	−0.133
0.700	−0.374
1.000	−0.580
1.500	−0.721
2.000	−0.934
3.000	−0.957
5.000	−1.000

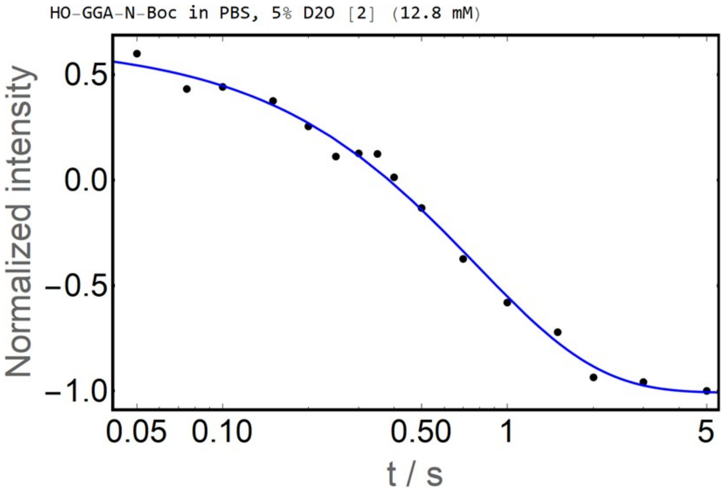

— $y = M1 \times [1 - \mathrm{Exp}(-t/\tau 1)] + c1$

	Estimate	Standard Error	t-Statistic	P-Value
M1	−1.65639	0.0367823	−45.0321	1.16046×10^{-15}
$\tau 1$	0.773078	0.0499553	15.4754	9.38887×10^{-10}
c1	0.648505	0.0298549	21.7219	1.33651×10^{-11}

Figure S6: Plot from the inversion recovery measurement of compound **1** in PBS including the delays with the respective normalized integrals and the obtained T1 value.

Data	
t	Integrals
0.050	−0.873
0.075	−0.841
0.100	−0.355
0.150	−0.544
0.200	−0.417
0.250	−0.280
0.300	−0.224
0.350	−0.056
0.400	−0.051
0.500	0.154
0.700	0.318
1.000	0.492
1.500	0.912
2.000	0.872
3.000	0.926
5.000	1.000

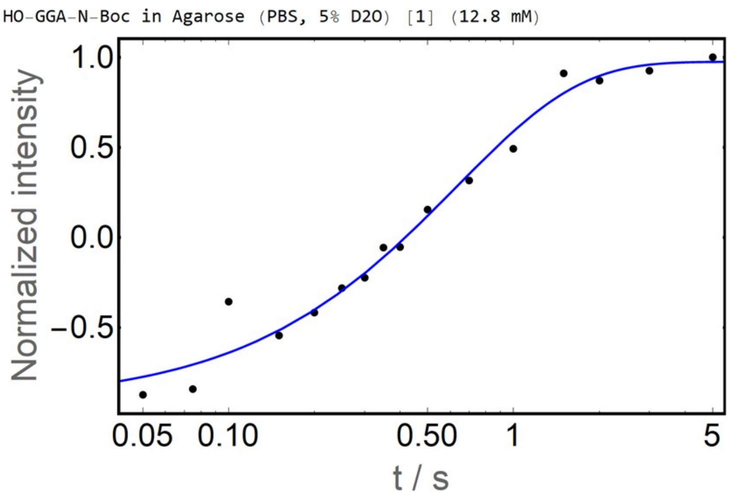

y=M1×[1−Exp(−t/τ1)]+c1

	Estimate	Standard Error	t-Statistic	P-Value
M1	1.89342	0.0822218	23.0282	6.36886×10^{-12}
τ1	0.628442	0.0754751	8.32648	1.43928×10^{-6}
c1	−0.91787	0.0723577	−12.6852	1.07065×10^{-8}

Figure S7: Plot from the inversion recovery measurement of compound **1** in Agarose gel including the delays with the respective normalized integrals and the obtained T1 value.

Data	
t	Integrals
0.050	−0.796
0.075	−0.836
0.100	−0.554
0.150	−0.356
0.200	−0.100
0.250	−0.022
0.300	0.037
0.350	0.074
0.400	0.094
0.500	0.136
0.700	0.227
1.000	0.320
1.500	0.464
2.000	0.578
3.000	0.750
5.000	1.000

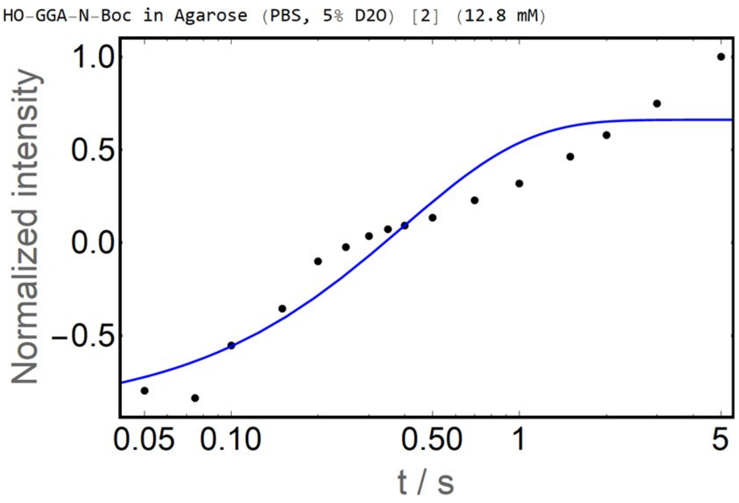

y=M1×[1−Exp(−t/τ1)]+c1

	Estimate	Standard Error	t-Statistic	P-Value
M1	1.5723	0.14625	10.7508	7.71923×10^{-8}
τ1	0.392677	0.0847806	4.63168	0.000469871
c1	−0.910649	0.141581	−6.43201	0.0000222842

Figure S8: Plot from the inversion recovery measurement of compound **1** in Agarose gel including the delays with the respective normalized integrals and the obtained T1 value.

Data	
t	Integrals
0.050	−0.659
0.075	−0.567
0.100	−0.476
0.150	−0.318
0.200	−0.172
0.250	−0.042
0.300	0.074
0.350	0.174
0.400	0.269
0.500	0.418
0.700	0.634
1.000	0.815
1.500	0.942
2.000	0.979
3.000	0.988
5.000	1.000

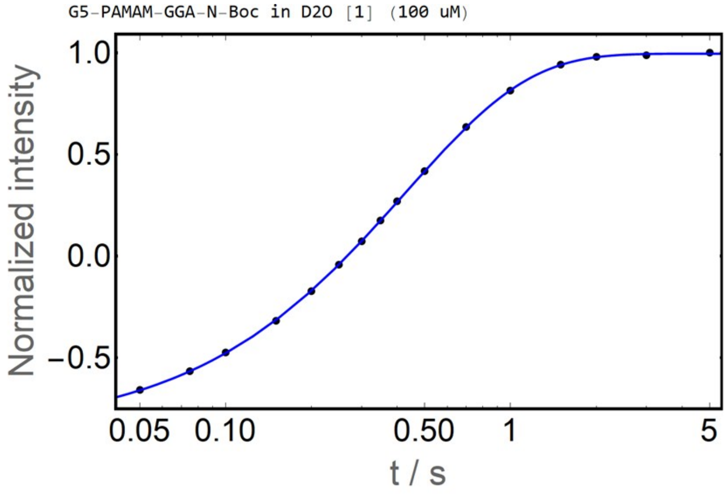

— y=M1×[1−Exp(−t/τ1)]+c1

	Estimate	Standard Error	t-Statistic	P-Value
M1	1.86174	0.00230646	807.185	6.11724×10^{-32}
τ1	0.427258	0.00126988	336.456	5.33203×10^{-27}
c1	−0.865968	0.0022061	−392.533	7.18851×10^{-28}

Figure S9: Plot from the inversion recovery measurement of compound **2** in D_2O including the delays with the respective normalized integrals and the obtained T1 value.

Data	
t	Integrals
0.050	−0.637
0.075	−0.548
0.100	−0.455
0.150	−0.294
0.200	−0.146
0.250	−0.023
0.300	0.093
0.350	0.192
0.400	0.284
0.500	0.431
0.700	0.646
1.000	0.827
1.500	0.948
2.000	0.982
3.000	1.000
5.000	0.995

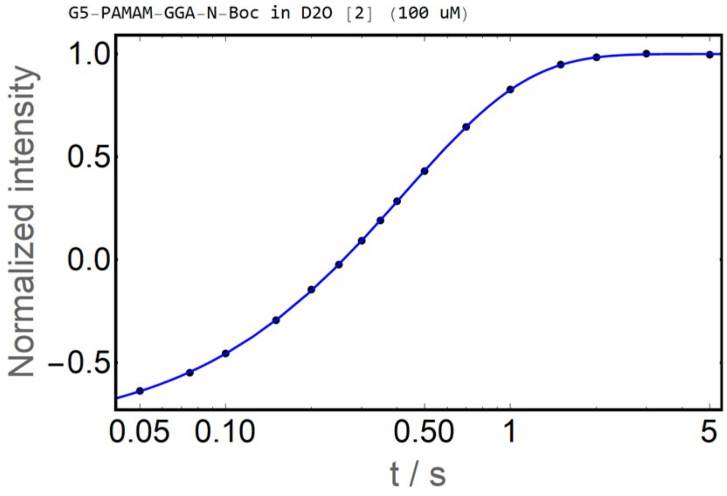

— y=M1×[1−Exp(−t/τ1)]+c1

	Estimate	Standard Error	t-Statistic	P-Value
M1	1.84304	0.00197139	934.896	9.06348×10^{-33}
τ1	0.423067	0.00108145	391.202	7.51305×10^{-28}
c1	−0.843985	0.00188839	−446.933	1.33024×10^{-28}

Figure S10: Plot from the inversion recovery measurement of compound **2** in D_2O including the delays with the respective normalized integrals and the obtained T1 value.

Data	
t	Integrals
0.050	−0.649
0.075	−0.158
0.100	−0.321
0.150	−0.192
0.200	0.065
0.250	0.071
0.300	0.413
0.350	0.263
0.400	0.364
0.500	0.310
0.700	0.476
1.000	0.619
1.500	1.000
2.000	0.556
3.000	0.712
5.000	0.840

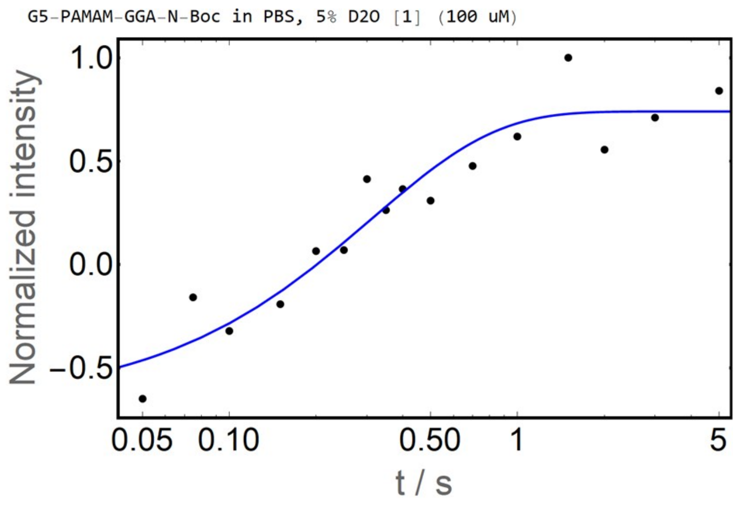

— y=M1×[1−Exp(−t/τ1)]+c1

	Estimate	Standard Error	t-Statistic	P-Value
M1	1.41308	0.144504	9.77882	2.3309×10^{-7}
τ1	0.311458	0.0677779	4.59527	0.000501999
c1	−0.672286	0.143876	−4.67267	0.000436243

Figure S11: Plot from the inversion recovery measurement of compound **2** in PBS including the delays with the respective normalized integrals and the obtained T1 value.

Data	
t	Integrals
0.050	−0.385
0.075	−0.496
0.100	−0.196
0.150	−0.115
0.200	0.059
0.250	0.089
0.300	0.134
0.350	0.190
0.400	0.297
0.500	0.347
0.700	0.565
1.000	0.658
1.500	0.856
2.000	1.000
3.000	0.708
5.000	0.960

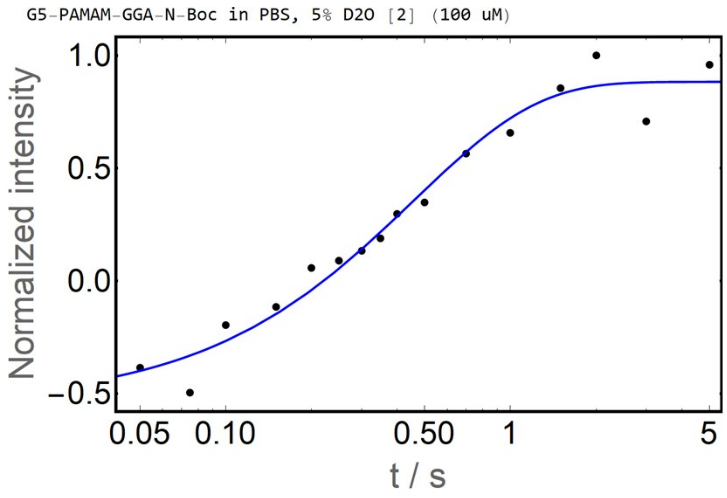

— y=M1×[1−Exp(−t/τ1)]+c1

	Estimate	Standard Error	t-Statistic	P-Value
M1	1.43041	0.0766553	18.6603	9.09367×10^{-11}
τ1	0.458121	0.0605372	7.5676	4.0866×10^{-6}
c1	−0.547048	0.0725132	−7.54412	4.22536×10^{-6}

Figure S12: Plot from the inversion recovery measurement of compound **2** in PBS including the delays with the respective normalized integrals and the obtained T1 value.

Data	
t	Integrals
0.050	0.327
0.075	−0.790
0.100	−0.290
0.150	−0.129
0.200	0.173
0.250	0.040
0.300	0.129
0.350	0.148
0.400	0.120
0.500	0.526
0.700	0.676
1.000	0.226
1.500	0.581
2.000	0.206
3.000	0.902
5.000	1.000

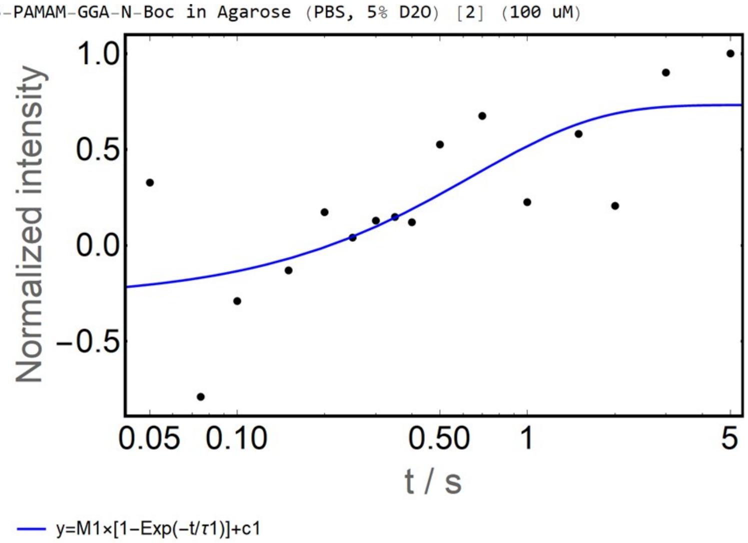

	Estimate	Standard Error	t-Statistic	P-Value
M1	1.01257	0.249622	4.05641	0.00135991
τ1	0.643524	0.441912	1.45623	0.169055
c1	−0.279868	0.218018	−1.28369	0.221659

Figure S13: Plot from the inversion recovery measurement of compound **2** in Agarose gel including the delays with the respective normalized integrals and the obtained T1 value.

Data	
t	Integrals
0.100	−0.721
0.300	−0.539
0.500	−0.344
0.700	−0.182
0.900	−0.044
1.200	0.137
1.500	0.271
2.000	0.484
3.000	0.714
5.000	0.914
6.500	0.968
8.000	0.980
10.000	1.000
15.000	1.000
20.000	0.986

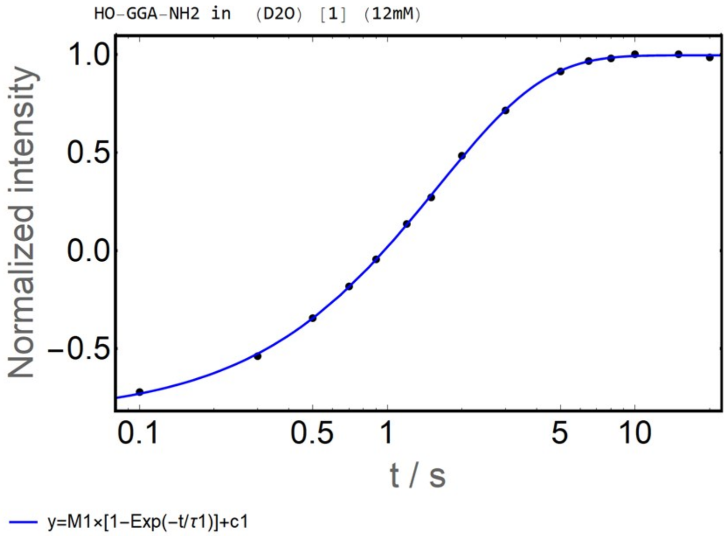

	Estimate	Standard Error	t-Statistic	P-Value
M1	1.83649	0.00672382	273.132	3.90434×10^{-24}
τ1	1.58764	0.0137536	115.435	1.19725×10^{-19}
c1	−0.84113	0.00649438	−129.517	3.01139×10^{-20}

Figure S14: Plot from the inversion recovery measurement of compound **3** in D_2O including the delays with the respective normalized integrals and the obtained T1 value.

Data	
t	Integrals
0.100	−0.762
0.300	−0.558
0.500	−0.377
0.700	−0.217
0.900	−0.077
1.200	0.108
1.500	0.253
2.000	0.448
3.000	0.696
5.000	0.907
6.500	0.957
8.000	0.983
10.000	0.988
15.000	0.994
20.000	0.999
30.000	1.000

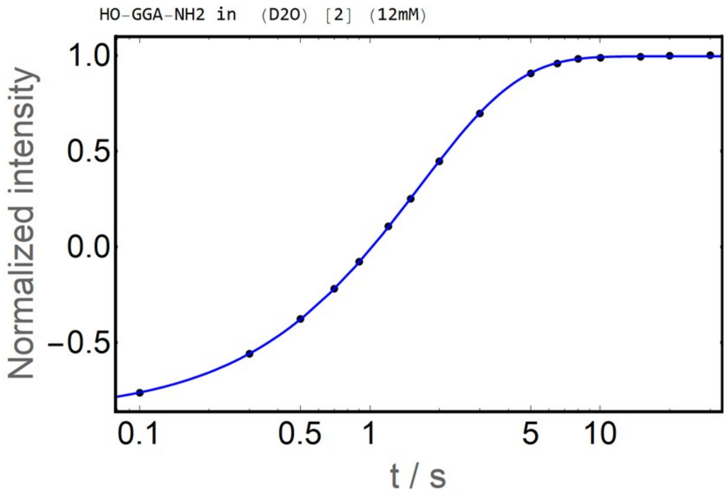

y=M1×[1−Exp(−t/τ1)]+c1

	Estimate	Standard Error	t-Statistic	P-Value
M1	1.86642	0.0026886	694.2	4.34395×10^{-31}
τ1	1.62687	0.00551007	295.254	2.91287×10^{-26}
c1	−0.871258	0.00260849	−334.009	5.86265×10^{-27}

Figure S15: Plot from the inversion recovery measurement of compound **3** in D_2O including the delays with the respective normalized integrals and the obtained T1 value.

Data	
t	Integrals
0.050	−0.765
0.075	−0.690
0.100	−0.821
0.150	−0.667
0.200	−0.642
0.250	−0.589
0.300	−0.585
0.350	−0.380
0.400	−0.302
0.500	−0.254
0.700	−0.188
1.000	0.012
1.500	0.337
2.000	0.370
3.000	0.910
5.000	1.000

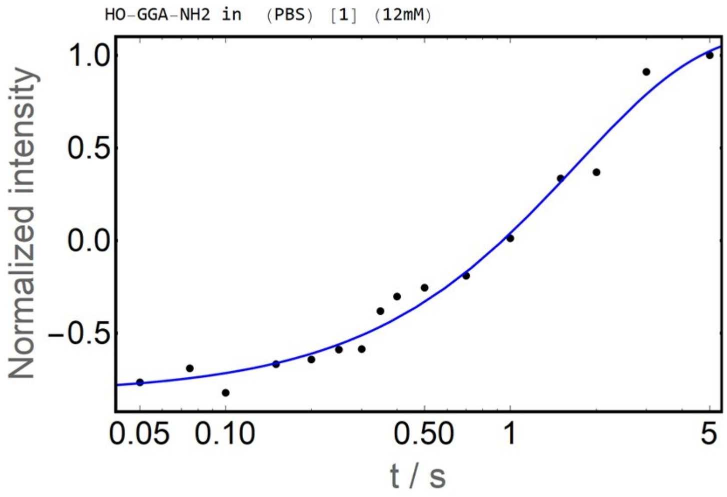

y=M1×[1−Exp(−t/τ1)]+c1

	Estimate	Standard Error	t-Statistic	P-Value
M1	1.95291	0.101601	19.2215	6.26481×10^{-11}
τ1	1.7061	0.23082	7.39147	5.25861×10^{-6}
c1	−0.825806	0.0400903	−20.5987	2.61714×10^{-11}

Figure S16: Plot from the inversion recovery measurement of compound **3** in PBS including the delays with the respective normalized integrals and the obtained T1 value.

Data	
t	Integrals
0.050	−0.771
0.075	−0.940
0.100	−0.791
0.150	−0.833
0.200	−0.675
0.250	−0.642
0.300	−0.541
0.350	−0.465
0.400	−0.428
0.500	−0.320
0.700	−0.115
1.000	0.091
1.500	0.380
2.000	0.577
3.000	0.952
5.000	1.000

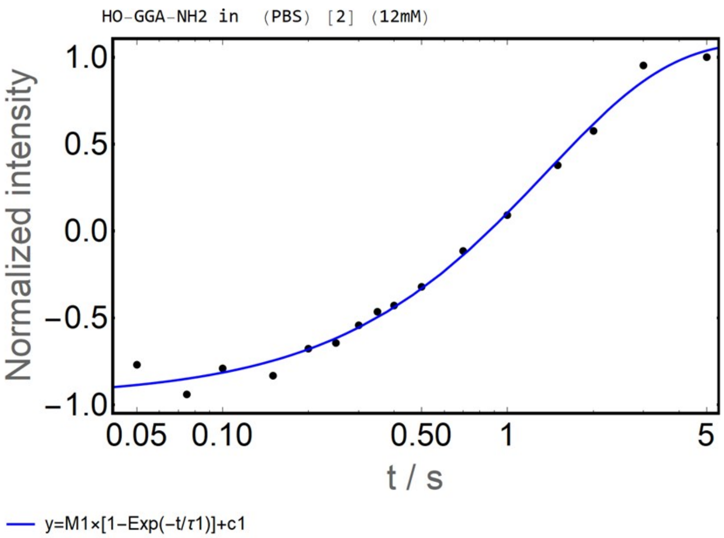

	Estimate	Standard Error	t-Statistic	P-Value
M1	2.05041	0.0582522	35.1989	2.78537×10^{-14}
τ1	1.36748	0.109222	12.5201	1.25416×10^{-8}
c1	−0.95893	0.0302506	−31.6996	1.07076×10^{-13}

Figure S17: Plot from the inversion recovery measurement of compound **3** in PBS including the delays with the respective normalized integrals and the obtained T1 value.

Data	
t	Integrals
0.050	−0.832
0.075	−0.897
0.100	−0.726
0.150	−0.868
0.200	−0.635
0.250	−0.572
0.300	−0.444
0.350	−0.432
0.400	−0.404
0.500	−0.305
0.700	−0.019
1.000	0.220
1.500	0.689
2.000	0.934
3.000	0.982
5.000	1.000

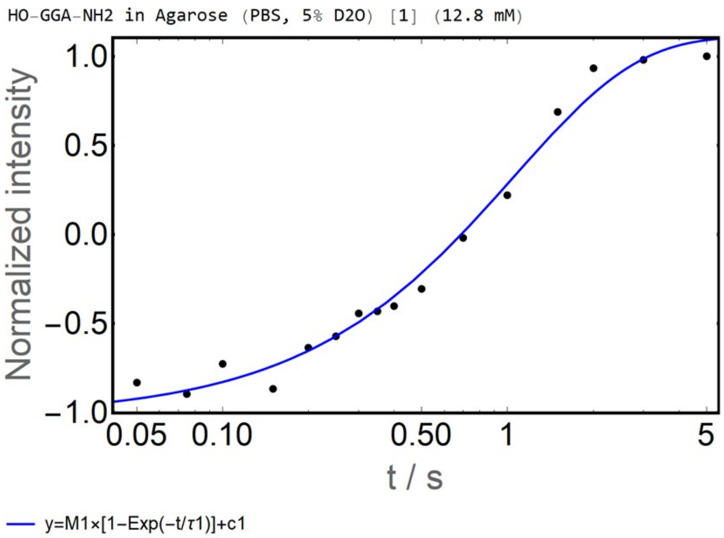

	Estimate	Standard Error	t-Statistic	P-Value
M1	2.13175	0.0717234	29.7218	2.44785×10^{-13}
τ1	1.05494	0.104742	10.0718	1.65587×10^{-7}
c1	−1.02146	0.0477475	−21.393	1.62147×10^{-11}

Figure S18: Plot from the inversion recovery measurement of compound **3** in Agarose gel including the delays with the respective normalized integrals and the obtained T1 value.

Data	
t	Integrals
0.050	−0.940
0.075	−0.648
0.100	−0.685
0.150	−0.548
0.200	−0.523
0.250	−0.465
0.300	−0.498
0.350	−0.370
0.400	−0.249
0.500	−0.268
0.700	−0.061
1.000	0.151
1.500	0.448
2.000	0.645
3.000	1.000
5.000	0.912

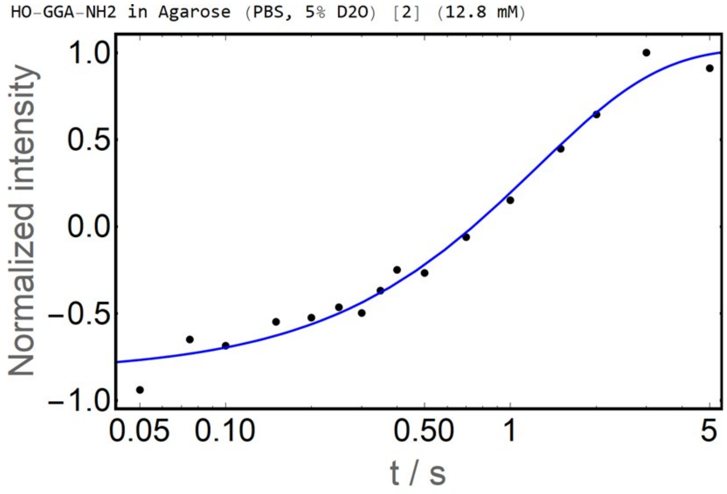

y=M1×[1−Exp(−t/τ1)]+c1

	Estimate	Standard Error	t-Statistic	P-Value
M1	1.86486	0.075806	24.6004	2.74787×10^{-12}
τ1	1.2318	0.144429	8.52879	1.10208×10^{-6}
c1	−0.841061	0.0439226	−19.1487	6.57106×10^{-11}

Figure S19: Plot from the inversion recovery measurement of compound **3** in Agarose gel including the delays with the respective normalized integrals and the obtained T1 value.

Data	
t	Integrals
0.050	−0.687
0.075	−0.599
0.100	−0.521
0.150	−0.375
0.200	−0.240
0.250	−0.123
0.300	−0.014
0.350	0.086
0.400	0.175
0.500	0.325
0.700	0.549
1.000	0.754
1.500	0.909
2.000	0.963
3.000	0.997
5.000	1.000

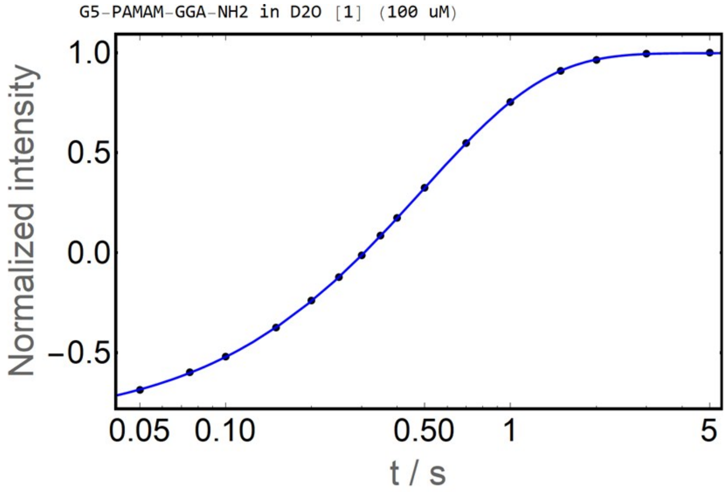

y=M1×[1−Exp(−t/τ1)]+c1

	Estimate	Standard Error	t-Statistic	P-Value
M1	1.86259	0.00148035	1258.21	1.90742×10^{-34}
τ1	0.491482	0.000989966	496.464	3.39296×10^{-29}
c1	−0.864244	0.001383	−624.906	1.70442×10^{-30}

Figure S20: Plot from the inversion recovery measurement of compound **4** in D_2O including the delays with the respective normalized integrals and the obtained T1 value.

Data	
t	Integrals
0.050	−0.654
0.075	−0.571
0.100	−0.497
0.150	−0.350
0.200	−0.221
0.250	−0.100
0.300	0.008
0.350	0.103
0.400	0.191
0.500	0.340
0.700	0.564
1.000	0.762
1.500	0.917
2.000	0.970
3.000	0.997
5.000	1.000

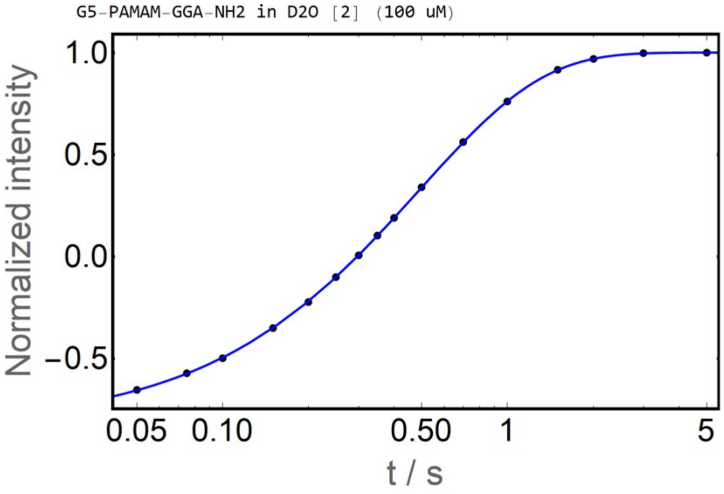

— y=M1×[1−Exp(−t/τ1)]+c1

	Estimate	Standard Error	t-Statistic	P-Value
M1	1.83481	0.00125047	1467.29	2.58551×10^{-35}
τ1	0.489884	0.000845079	579.691	4.52482×10^{-30}
c1	−0.833526	0.00116895	−713.053	3.06623×10^{-31}

Figure S21: Plot from the inversion recovery measurement of compound **4** in D_2O including the delays with the respective normalized integrals and the obtained T1 value.

Data	
t	Integrals
0.050	−0.464
0.075	−0.214
0.100	−0.204
0.150	−0.025
0.200	0.008
0.250	0.078
0.300	0.406
0.350	0.238
0.400	0.380
0.500	0.455
0.700	0.660
1.000	0.549
1.500	1.000
2.000	0.725
3.000	0.777
5.000	0.833

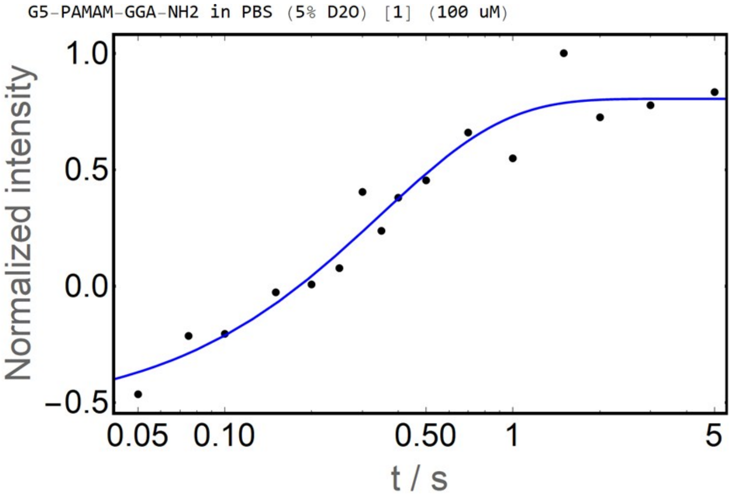

— y=M1×[1−Exp(−t/τ1)]+c1

	Estimate	Standard Error	t-Statistic	P-Value
M1	1.35646	0.0973928	13.9278	3.43437×10^{-9}
τ1	0.346692	0.0550951	6.29262	0.0000277693
c1	−0.551487	0.0957872	−5.75742	0.0000662743

Figure S22: Plot from the inversion recovery measurement of compound **4** in PBS including the delays with the respective normalized integrals and the obtained T1 value.

Data	
t	Integrals
0.050	−0.422
0.075	−0.411
0.100	−0.223
0.150	−0.129
0.200	−0.057
0.250	0.143
0.300	0.336
0.350	0.317
0.400	0.404
0.500	0.574
0.700	0.766
1.000	0.878
1.500	0.940
2.000	0.950
3.000	0.931
5.000	1.000

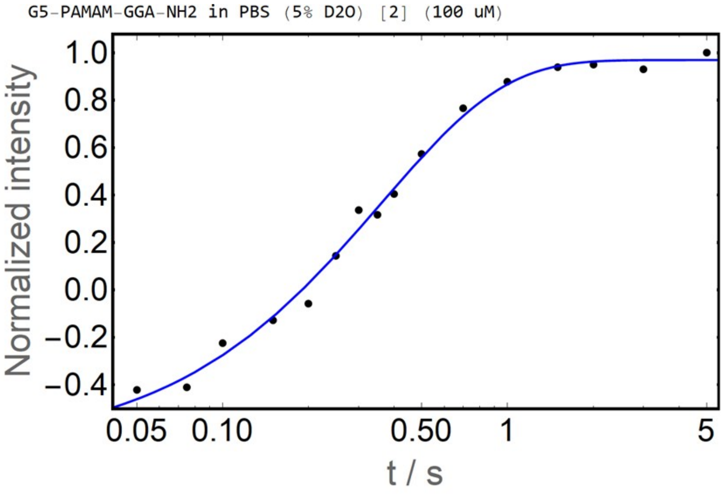

y=M1×[1−Exp(−t/τ1)]+c1

	Estimate	Standard Error	t-Statistic	P-Value
M1	1.64349	0.0404691	40.611	4.40753×10^{-15}
τ1	0.360175	0.0199141	18.0864	1.3458×10^{-10}
c1	−0.67395	0.0396175	−17.0114	2.89626×10^{-10}

Figure S23: Plot from the inversion recovery measurement of compound **4** in PBS including the delays with the respective normalized integrals and the obtained T1 value.

Data	
t	Integrals
0.050	−0.283
0.075	−0.831
0.100	−0.584
0.150	0.009
0.200	0.063
0.250	−0.149
0.300	0.002
0.350	0.125
0.400	0.089
0.500	0.452
0.700	0.536
1.000	0.792
1.500	0.949
2.000	1.000
3.000	0.956
5.000	0.980

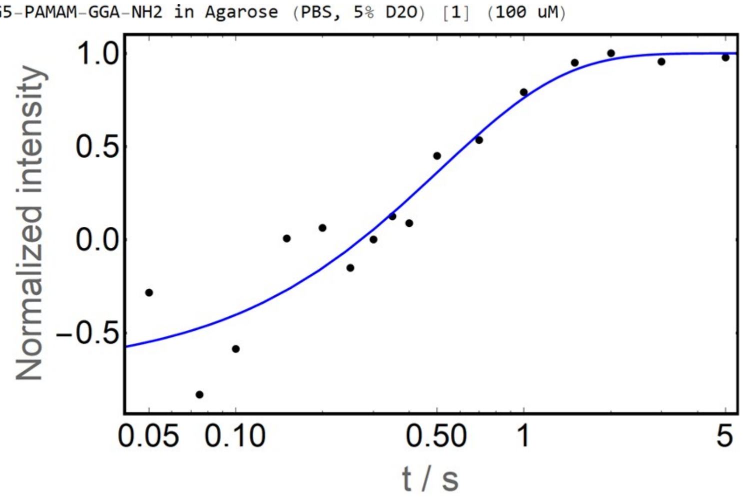

y=M1×[1−Exp(−t/τ1)]+c1

	Estimate	Standard Error	t-Statistic	P-Value
M1	1.70769	0.144055	11.8544	2.41898×10^{-8}
τ1	0.508058	0.11001	4.61831	0.000481414
c1	−0.706946	0.133716	−5.28692	0.000147155

Figure S24: Plot from the inversion recovery measurement of compound **4** in Agarose gel including the delays with the respective normalized integrals and the obtained T1 value.

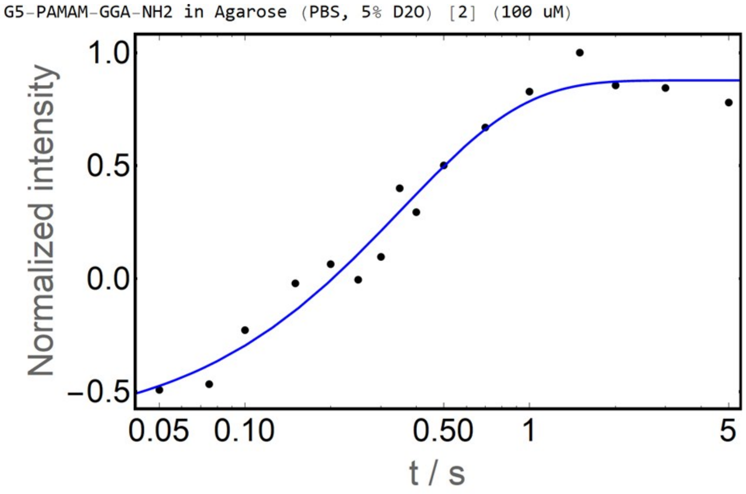

Data	
t	Integrals
0.050	−0.493
0.075	−0.468
0.100	−0.228
0.150	−0.021
0.200	0.065
0.250	−0.005
0.300	0.098
0.350	0.402
0.400	0.294
0.500	0.501
0.700	0.670
1.000	0.829
1.500	1.000
2.000	0.856
3.000	0.843
5.000	0.780

y=M1×[1−Exp(−t/τ1)]+c1

	Estimate	Standard Error	t-Statistic	P-Value
M1	1.55789	0.0839011	18.5682	9.67663×10^{-11}
τ1	0.3539	0.0425126	8.32459	1.44291×10^{-6}
c1	−0.679757	0.0823131	−8.25818	1.57666×10^{-6}

Figure S25: Plot from the inversion recovery measurement of compound **4** in Agarose gel including the delays with the respective normalized integrals and the obtained T1 value.

T_S measurements

T_S measurements have been performed using the SLIC sequence, as discussed in the main article. The obtained plots are listed below. The values given in the main article are the mean values taken from the values obtained from two different samples. The duration and power of the SLIC pulses where optimized for each sample. The singlet evolution time has been 200 ms in all cases.

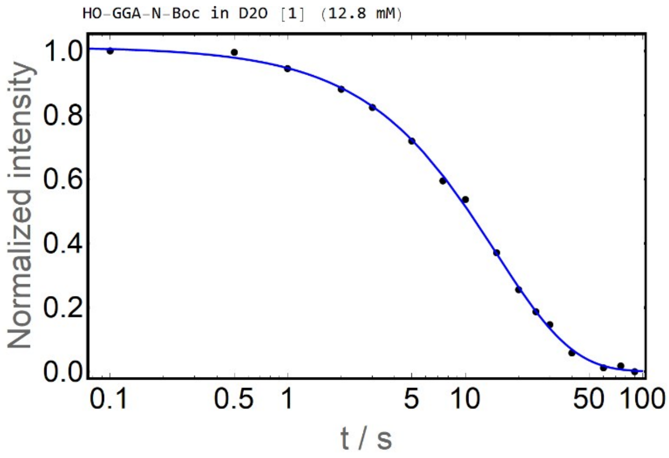

Data	
t	Integrals
0.100	1.000
0.500	0.996
1.000	0.945
2.000	0.881
3.000	0.824
5.000	0.719
7.500	0.596
10.000	0.537
15.000	0.371
20.000	0.256
25.000	0.187
30.000	0.146
40.000	0.059
60.000	0.012
75.000	0.018
90.000	0.001

y=M1×[1−Exp(−t/τ1)]+c1

	Estimate	Standard Error	t-Statistic	P-Value
M1	−1.01209	0.00734568	−137.78	5.8348×10^{-22}
τ1	14.8291	0.305788	48.4946	4.45367×10^{-16}
c1	1.0128	0.00557303	181.733	1.59802×10^{-23}

Figure S26: Plot from the Ts measurement of compound **1** in D_2O including the delays with the respective normalized integrals and the obtained Ts value.

Data	
t	Integrals
0.100	1.000
0.500	0.939
1.000	0.905
2.000	0.824
3.000	0.751
5.000	0.635
7.500	0.524
10.000	0.407
15.000	0.257
20.000	0.158
25.000	0.129
30.000	0.075
40.000	0.027
60.000	−0.007
75.000	−0.008
90.000	−0.009

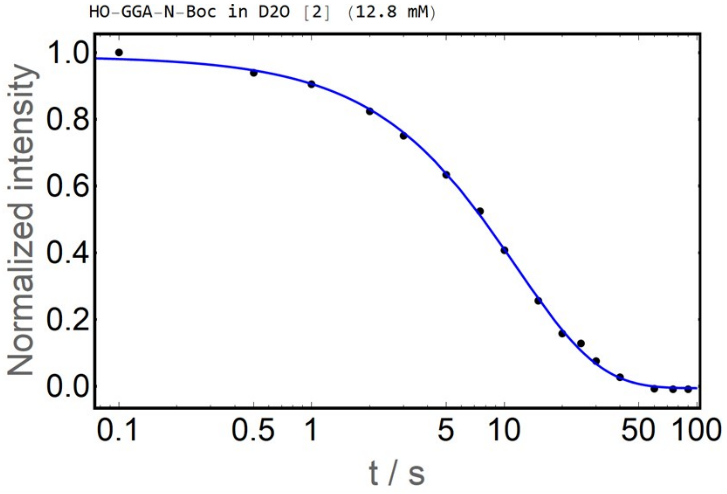

y=M1×[1−Exp(−t/τ1)]+c1

	Estimate	Standard Error	t-Statistic	P-Value
M1	−0.995022	0.0072945	−136.407	6.64563×10^{-22}
τ1	11.4848	0.241108	47.6336	5.61477×10^{-16}
c1	0.989292	0.00602661	164.154	5.99397×10^{-23}

Figure S27: Plot from the Ts measurement of compound **1** in D_2O including the delays with the respective normalized integrals and the obtained Ts value.

Data	
t	Integrals
0.100	1.000
0.300	0.957
0.500	0.856
0.700	0.881
0.900	0.795
1.200	0.738
1.500	0.704
2.000	0.603
3.000	0.451
5.000	0.299
6.500	0.164
8.000	0.110
10.000	0.058
15.000	0.006
20.000	0.006
30.000	−0.004

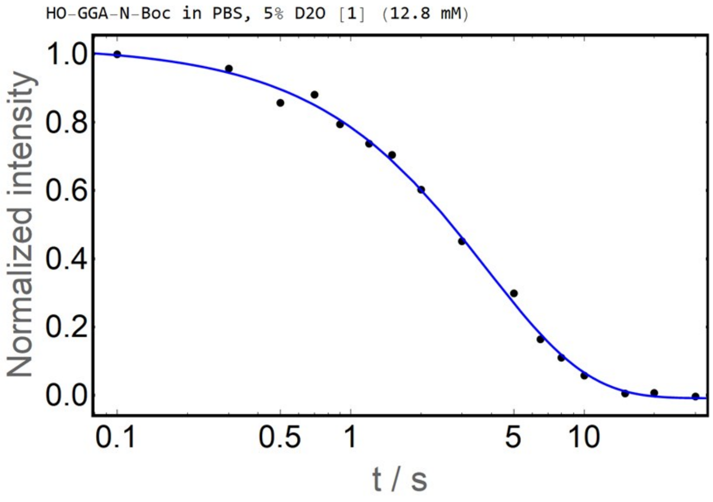

y=M1×[1−Exp(−t/τ1)]+c1

	Estimate	Standard Error	t-Statistic	P-Value
M1	−1.03241	0.0132067	−78.1732	9.16014×10^{-19}
τ1	3.81659	0.149193	25.5815	1.6687×10^{-12}
c1	1.02346	0.0110729	92.4292	1.04167×10^{-19}

Figure S28: Plot from the Ts measurement of compound **1** in PBS including the delays with the respective normalized integrals and the obtained Ts value.

Data	
t	Integrals
0.100	1.000
0.300	0.873
0.500	0.888
0.700	0.862
0.900	0.797
1.200	0.723
1.500	0.682
2.000	0.543
3.000	0.489
5.000	0.292
6.500	0.204
8.000	0.165
10.000	0.064
15.000	0.032
20.000	0.014
30.000	0.003

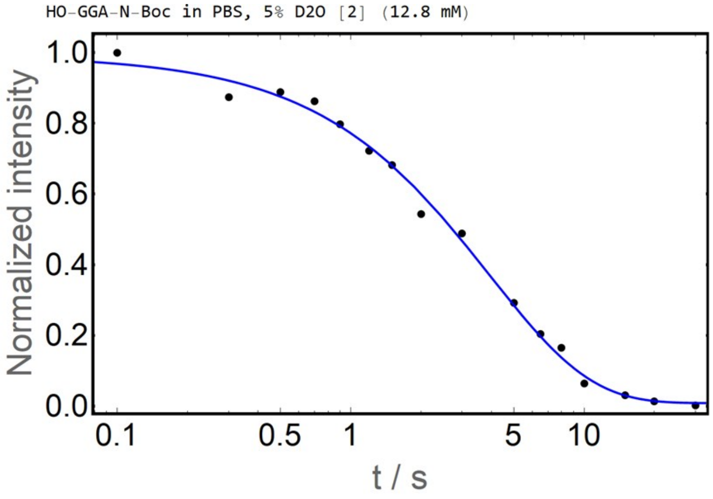

y=M1×[1−Exp(−t/τ1)]+c1

	Estimate	Standard Error	t-Statistic	P-Value
M1	−0.984462	0.0191044	−51.5306	2.0302×10^{-16}
τ1	3.93228	0.233291	16.8557	3.24819×10^{-10}
c1	0.992983	0.0158134	62.7938	1.56914×10^{-17}

Figure S29: Plot from the Ts measurement of compound **1** in PBS including the delays with the respective normalized integrals and the obtained Ts value.

Data	
t	Integrals
0.100	1.000
0.300	0.933
0.500	0.930
0.700	0.831
0.900	0.687
1.200	0.684
1.500	0.622
2.000	0.452
3.000	0.481
5.000	0.147
6.500	0.097
8.000	0.096
10.000	0.032
15.000	0.005
20.000	0.019
30.000	−0.024

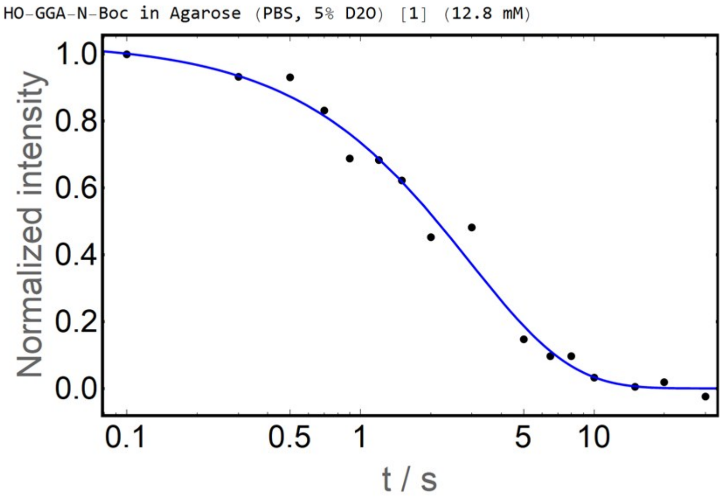

y=M1×[1−Exp(−t/τ1)]+c1

	Estimate	Standard Error	t-Statistic	P-Value
M1	−1.03582	0.0342933	−30.2048	1.99061×10^{-13}
τ1	2.91566	0.284817	10.237	1.37022×10^{-7}
c1	1.03625	0.0313776	33.025	6.32537×10^{-14}

Figure S30: Plot from the Ts measurement of compound **1** in Agarose gel including the delays with the respective normalized integrals and the obtained Ts value.

Data	
t	Integrals
0.100	1.000
0.300	0.993
0.500	0.845
0.700	0.932
0.900	0.852
1.200	0.614
1.500	0.643
2.000	0.588
3.000	0.440
5.000	0.203
6.500	0.185
8.000	0.136
10.000	0.017
15.000	0.045
20.000	−0.046
30.000	−0.007

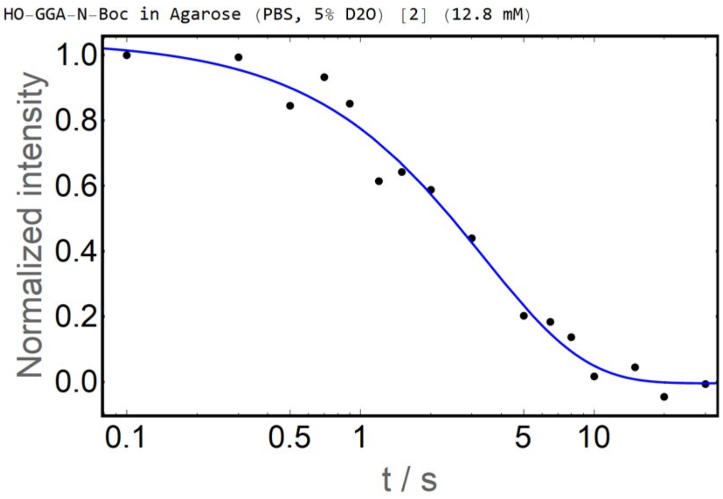

y=M1×[1−Exp(−t/τ1)]+c1

	Estimate	Standard Error	t-Statistic	P-Value
M1	−1.04874	0.0379515	−27.6336	6.22434×10^{-13}
τ1	3.36464	0.368708	9.12548	5.14651×10^{-7}
c1	1.0447	0.0333606	31.3154	1.25226×10^{-13}

Figure S31: Plot from the Ts measurement of compound **1** in Agarose gel including the delays with the respective normalized integrals and the obtained Ts value.

Data	
t	Integrals
0.100	1.000
0.300	0.869
0.500	0.804
0.700	0.824
0.900	0.902
1.200	0.868
1.500	0.746
2.000	0.759
3.000	0.677
5.000	0.518
6.500	0.590
8.000	0.481
10.000	0.272
15.000	0.180
20.000	0.143
30.000	0.106

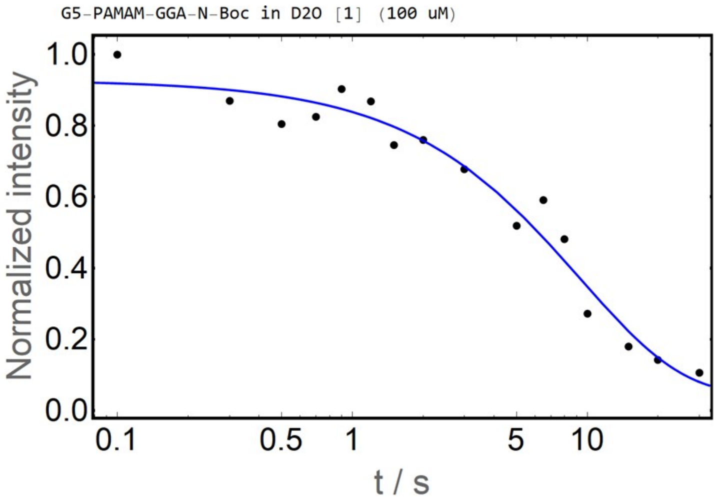

y=M1×[1−Exp(−t/τ1)]+c1

	Estimate	Standard Error	t-Statistic	P-Value
M1	−0.883579	0.06576	−13.4364	5.32503×10^{-9}
τ1	9.35067	1.79986	5.19521	0.000172524
c1	0.928013	0.0278837	33.2815	5.72631×10^{-14}

Figure S32: Plot from the Ts measurement of compound **2** in D_2O including the delays with the respective normalized integrals and the obtained Ts value.

Data	
t	Integrals
0.100	1.000
0.500	0.945
1.000	0.936
1.500	0.871
2.000	0.817
3.000	0.735
5.000	0.607
7.500	0.503
10.000	0.484
12.500	0.309
15.000	0.283
20.000	0.112
25.000	0.144
30.000	0.110
40.000	−0.020
60.000	0.036

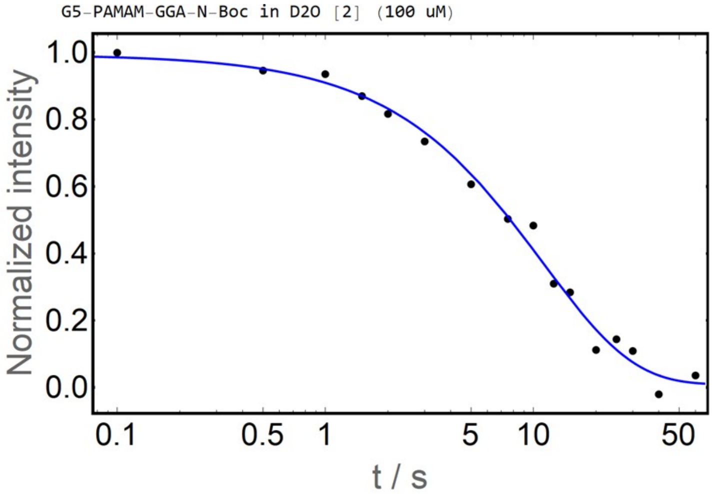

y=M1×[1−Exp(−t/τ1)]+c1

	Estimate	Standard Error	t-Statistic	P-Value
M1	−0.985423	0.0285223	−34.5493	3.54008×10^{-14}
τ1	11.1198	0.92053	12.0797	1.93009×10^{-8}
c1	0.994412	0.020081	49.52	3.39768×10^{-16}

Figure S33: Plot from the Ts measurement of compound **2** in D_2O including the delays with the respective normalized integrals and the obtained Ts value.

Data	
t	Integrals
0.100	1.000
1.000	0.976
2.000	0.931
3.000	0.907
5.000	0.847
7.500	0.798
10.000	0.763
12.500	0.672
15.000	0.622
20.000	0.549
25.000	0.455
30.000	0.370
40.000	0.282
60.000	0.125
90.000	0.061
120.000	−0.002

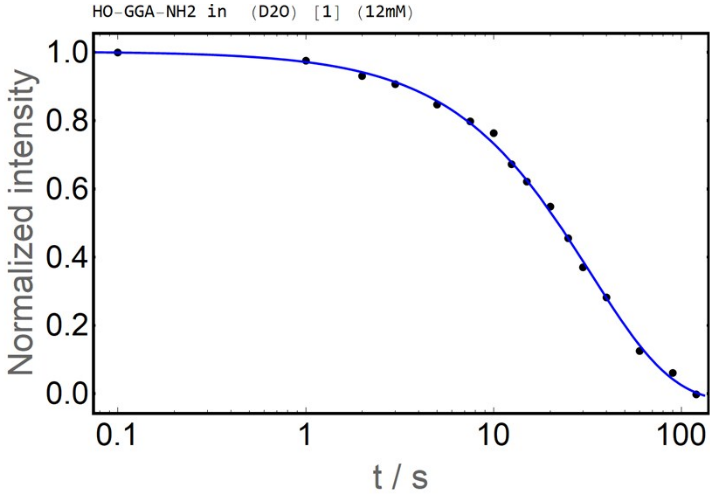

y=M1×[1−Exp(−t/τ1)]+c1

	Estimate	Standard Error	t-Statistic	P-Value
M1	−1.02582	0.012529	−81.8756	5.02511×10^{-19}
τ1	32.7931	1.04736	31.3104	1.25485×10^{-13}
c1	1.00243	0.00685229	146.291	2.67818×10^{-22}

Figure S34: Plot from the Ts measurement of compound **3** in D_2O including the delays with the respective normalized integrals and the obtained Ts value.

Data	
t	Integrals
0.100	1.000
1.000	0.929
2.000	0.911
3.000	0.861
5.000	0.799
7.500	0.746
10.000	0.676
12.500	0.598
15.000	0.550
20.000	0.470
25.000	0.391
30.000	0.324
40.000	0.227
60.000	0.109
90.000	0.027
120.000	0.002

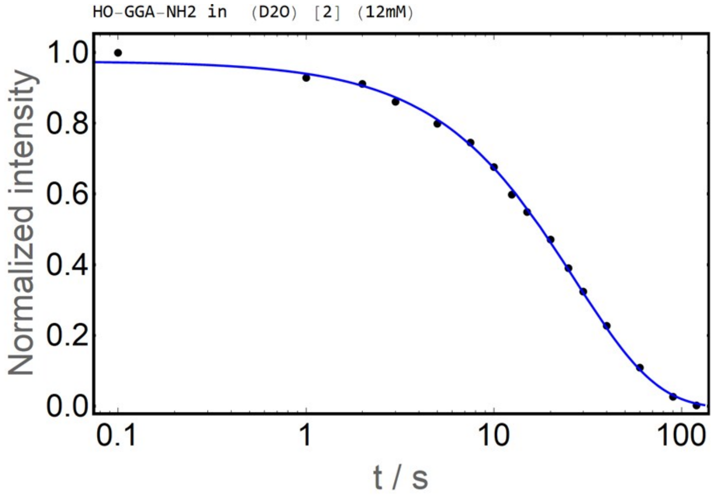

— y=M1×[1−Exp(−t/τ1)]+c1

	Estimate	Standard Error	t-Statistic	P-Value
M1	−0.980093	0.00985634	−99.4378	4.03297×10^{-20}
τ1	27.117	0.716727	37.8345	1.09874×10^{-14}
c1	0.975733	0.00619889	157.404	1.03432×10^{-22}

Figure S35: Plot from the Ts measurement of compound **3** in D_2O including the delays with the respective normalized integrals and the obtained Ts value.

Data	
t	Integrals
0.100	1.000
0.500	0.982
1.000	0.844
1.500	0.747
2.000	0.751
3.000	0.647
5.000	0.468
7.500	0.291
10.000	0.210
12.500	0.186
15.000	0.108
20.000	0.050
25.000	−0.043
30.000	−0.046
40.000	−0.028
60.000	−0.068

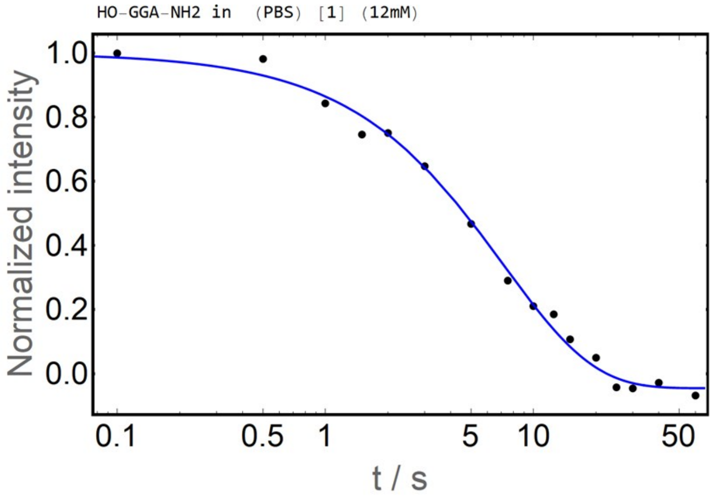

— y=M1×[1−Exp(−t/τ1)]+c1

	Estimate	Standard Error	t-Statistic	P-Value
M1	−1.04613	0.0221871	−47.1504	6.40592×10^{-16}
τ1	7.17292	0.446287	16.0724	5.87223×10^{-10}
c1	1.00076	0.0191073	52.3757	1.64479×10^{-16}

Figure S36: Plot from the Ts measurement of compound **3** in PBS including the delays with the respective normalized integrals and the obtained Ts value.

Data	
t	Integrals
0.100	1.000
0.500	0.917
1.000	0.882
1.500	0.829
2.000	0.780
3.000	0.697
5.000	0.553
7.500	0.421
10.000	0.319
12.500	0.244
15.000	0.191
20.000	0.118
25.000	0.059
30.000	0.044
40.000	0.034
60.000	0.017

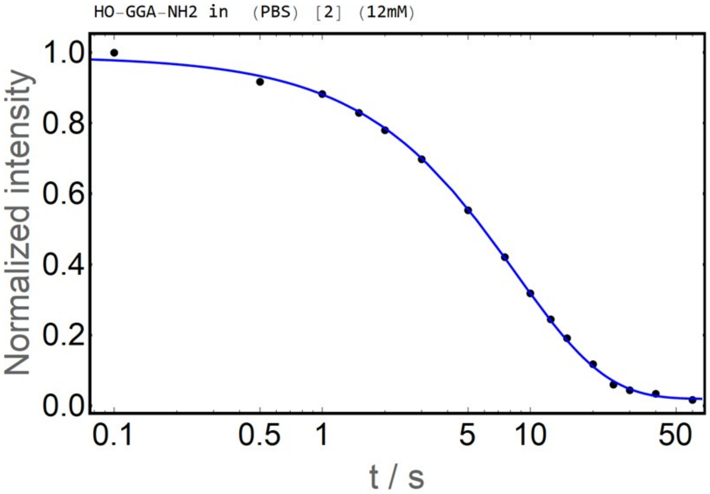

y=M1×[1−Exp(−t/τ1)]+c1

	Estimate	Standard Error	t-Statistic	P-Value
M1	−0.969821	0.00646791	−149.943	1.9439×10^{-22}
τ1	8.4701	0.165478	51.1856	2.21461×10^{-16}
c1	0.989161	0.00524056	188.751	9.7663×10^{-24}

Figure S37: Plot from the Ts measurement of compound **3** in PBS including the delays with the respective normalized integrals and the obtained Ts value.

Data	
t	Integrals
0.100	0.980
0.500	1.000
1.000	0.958
1.500	0.919
2.000	0.795
3.000	0.756
5.000	0.553
7.500	0.402
10.000	0.341
12.500	0.214
15.000	0.158
20.000	0.091
25.000	0.045
30.000	0.011
40.000	−0.006
60.000	−0.016

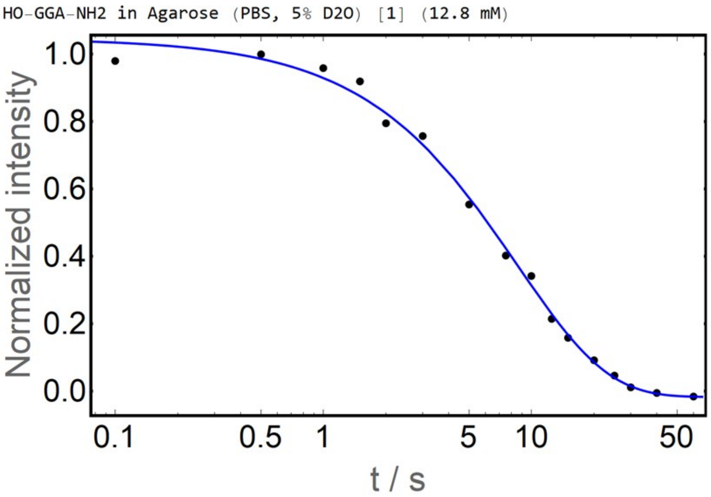

y=M1×[1−Exp(−t/τ1)]+c1

	Estimate	Standard Error	t-Statistic	P-Value
M1	−1.06412	0.0190841	−55.7596	7.31312×10^{-17}
τ1	8.5386	0.448446	19.0404	7.05719×10^{-11}
c1	1.04665	0.0154104	67.9186	5.67525×10^{-18}

Figure S38: Plot from the Ts measurement of compound **3** in Agarose gel including the delays with the respective normalized integrals and the obtained Ts value.

Data	
t	Integrals
0.100	1.000
0.500	0.982
1.000	0.919
1.500	0.870
2.000	0.807
3.000	0.746
5.000	0.575
7.500	0.428
10.000	0.307
12.500	0.250
15.000	0.174
20.000	0.099
25.000	0.078
30.000	0.026
40.000	−0.006
60.000	0.004

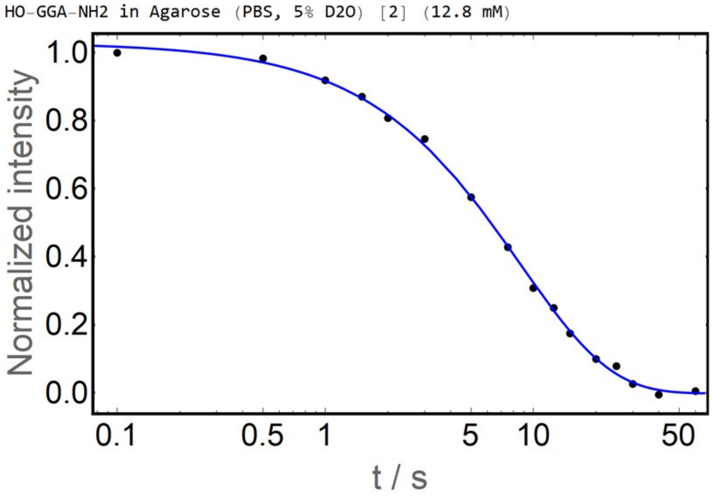

y=M1×[1−Exp(−t/τ1)]+c1

	Estimate	Standard Error	t-Statistic	P-Value
M1	−1.03113	0.00877721	−117.478	4.62743×10^{-21}
τ1	8.6578	0.215694	40.1394	5.12468×10^{-15}
c1	1.02906	0.00704569	146.056	2.73474×10^{-22}

Figure S39: Plot from the Ts measurement of compound **3** in Agarose gel including the delays with the respective normalized integrals and the obtained Ts value.

Data	
t	Integrals
0.100	1.000
0.300	0.983
0.500	0.987
0.700	0.902
0.900	0.867
1.200	0.826
1.500	0.812
2.000	0.761
3.000	0.614
5.000	0.507
6.500	0.383
8.000	0.326
10.000	0.241
15.000	0.091
20.000	0.099
30.000	0.038

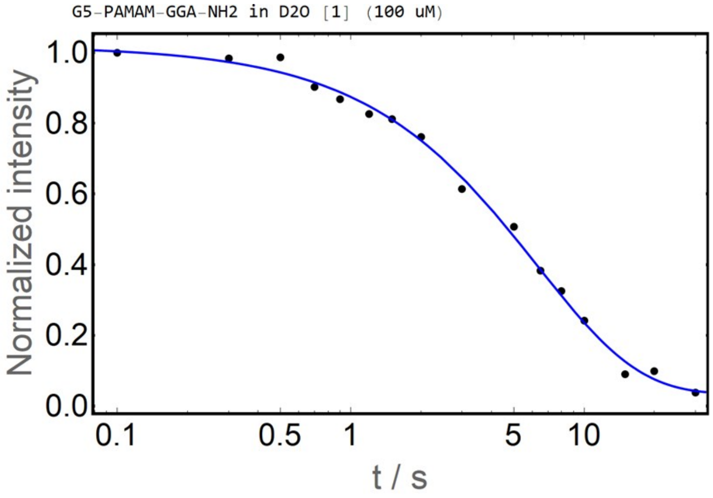

y=M1×[1−Exp(−t/τ1)]+c1

	Estimate	Standard Error	t-Statistic	P-Value
M1	−0.984483	0.0186035	−52.9192	1.43907×10^{-16}
τ1	6.31015	0.345104	18.2848	1.17375×10^{-10}
c1	1.01884	0.0115754	88.0172	1.96543×10^{-19}

Figure S40: Plot from the Ts measurement of compound **4** in D_2O including the delays with the respective normalized integrals and the obtained Ts value.

Data	
t	Integrals
0.100	0.988
0.300	0.995
0.500	0.945
0.700	1.000
0.900	0.866
1.200	0.895
1.500	0.823
2.000	0.836
3.000	0.692
5.000	0.533
6.500	0.446
8.000	0.311
10.000	0.262
15.000	0.157
20.000	0.040
30.000	0.045

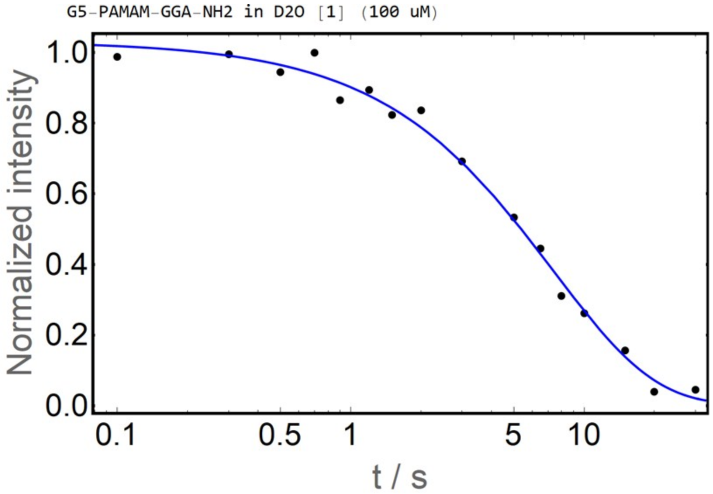

y=M1×[1−Exp(−t/τ1)]+c1

	Estimate	Standard Error	t-Statistic	P-Value
M1	−1.02863	0.0293733	−35.0192	2.97502×10^{-14}
τ1	7.38229	0.587719	12.5609	1.20587×10^{-8}
c1	1.03211	0.0159891	64.5504	1.09738×10^{-17}

Figure S41: Plot from the Ts measurement of compound **4** in D_2O without decoupling including the delays with the respective normalized integrals and the obtained Ts value.

Data	
t	Integrals
0.100	0.987
0.300	1.000
0.500	0.988
0.700	0.950
0.900	0.905
1.200	0.860
1.500	0.864
2.000	0.762
3.000	0.697
5.000	0.542
6.500	0.423
8.000	0.362
10.000	0.243
15.000	0.120
20.000	0.061
30.000	0.008

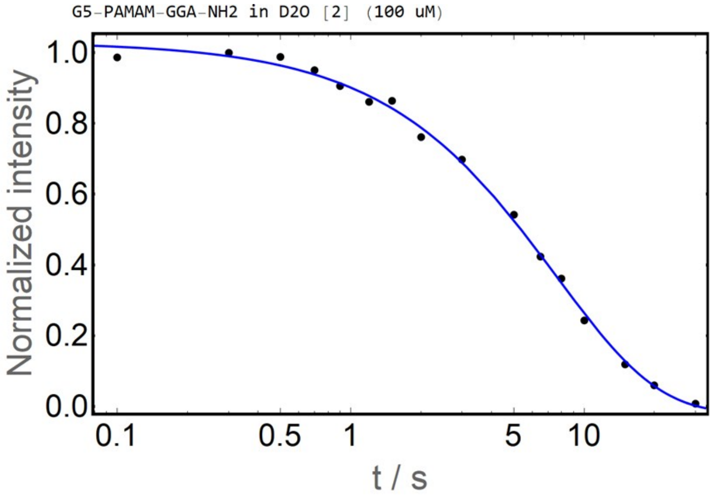

y=M1×[1−Exp(−t/τ1)]+c1

	Estimate	Standard Error	t-Statistic	P-Value
M1	−1.04855	0.0163915	−63.9689	1.23397×10^{-17}
τ1	7.6132	0.329014	23.1395	5.99034×10^{-12}
c1	1.03029	0.00866688	118.877	3.968×10^{-21}

Figure S42: Plot from the Ts measurement of compound **4** in D_2O including the delays with the respective normalized integrals and the obtained Ts value.

Data	
t	Integrals
0.100	1.000
0.300	0.904
0.500	0.773
0.700	0.902
0.900	0.835
1.200	0.866
1.500	0.796
2.000	0.691
3.000	0.600
5.000	0.507
6.500	0.306
8.000	0.206
10.000	0.220
15.000	0.090
20.000	−0.003
30.000	0.025

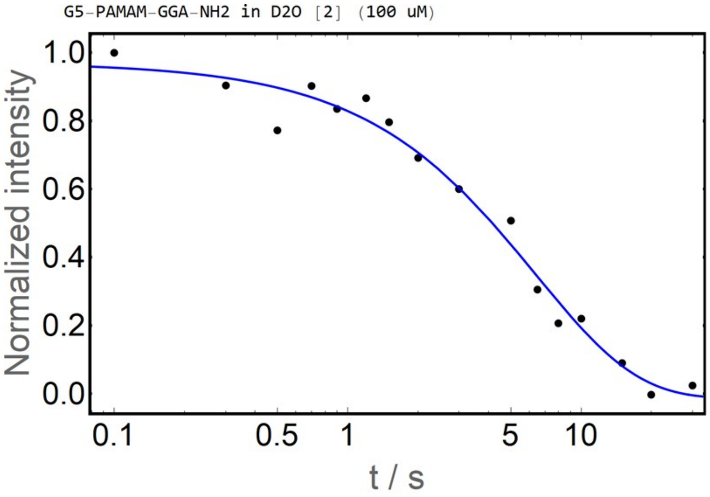

— y=M1×[1−Exp(−t/τ1)]+c1

	Estimate	Standard Error	t-Statistic	P-Value
M1	−0.984285	0.0433391	−22.7112	7.59527×10^{-12}
τ1	6.40445	0.813576	7.87197	2.66697×10^{-6}
c1	0.970957	0.0266535	36.4288	1.78972×10^{-14}

Figure S43: Plot from the Ts measurement of compound **4** in D_2O without decoupling including the delays with the respective normalized integrals and the obtained Ts value.

Data	
t	Integrals
0.100	1.000
0.300	0.869
0.500	0.736
0.700	0.597
0.900	0.660
1.200	0.544
1.500	0.456
2.000	0.298
2.500	0.237
3.000	0.217
4.000	0.126
5.000	0.078
6.500	0.011
8.000	−0.097
10.000	−0.049
15.000	−0.042

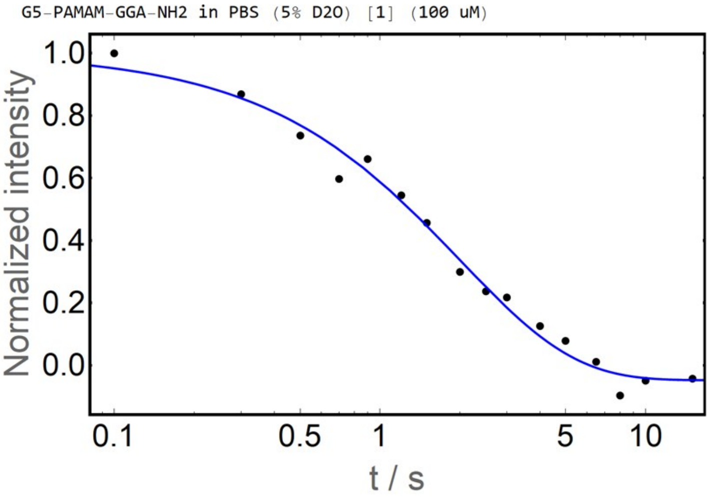

— y=M1×[1−Exp(−t/τ1)]+c1

	Estimate	Standard Error	t-Statistic	P-Value
M1	−1.05037	0.0347383	−30.2368	1.9638×10^{-13}
τ1	1.99106	0.171488	11.6105	3.10117×10^{-8}
c1	1.00257	0.0328102	30.5566	1.71576×10^{-13}

Figure S44: Plot from the Ts measurement of compound **4** in PBS including the delays with the respective normalized integrals and the obtained Ts value.

Data	
t	Integrals
0.100	1.000
0.300	0.831
0.500	0.765
0.700	0.713
0.900	0.593
1.200	0.512
1.500	0.399
2.000	0.366
2.500	0.214
3.000	0.124
4.000	0.081
5.000	0.069
6.500	0.040
8.000	0.007
10.000	0.005
15.000	−0.002

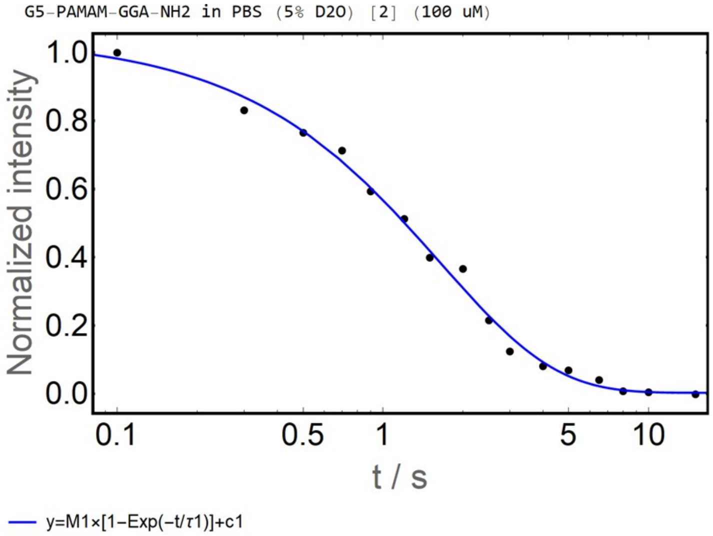

	Estimate	Standard Error	t-Statistic	P-Value
M1	−1.04115	0.0226691	−45.9283	8.99556×10^{-16}
τ1	1.63436	0.086762	18.8372	8.07656×10^{-11}
c1	1.04393	0.0220815	47.2761	6.18919×10^{-16}

Figure S45: Plot from the Ts measurement of compound **4** in PBS including the delays with the respective normalized integrals and the obtained Ts value.

Data	
t	Integrals
0.100	1.000
0.300	0.743
0.500	0.767
0.700	0.783
0.900	0.558
1.200	0.332
1.500	0.380
2.000	0.424
2.500	0.130
3.000	0.151
4.000	0.081
5.000	0.063
6.500	0.042
8.000	0.058
10.000	0.113
15.000	−0.025

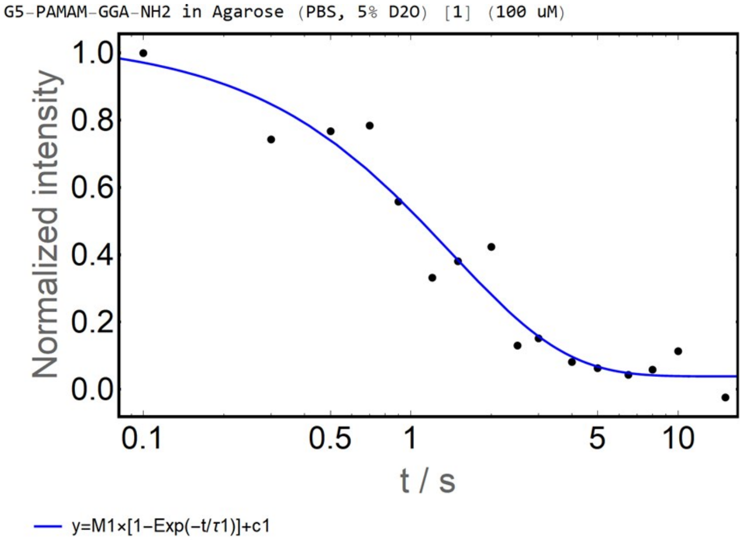

	Estimate	Standard Error	t-Statistic	P-Value
M1	−1.00136	0.0704406	−14.2157	2.67277×10^{-9}
τ1	1.4092	0.229752	6.13358	0.000035811
c1	1.03968	0.0696479	14.9276	1.46542×10^{-9}

Figure S46: Plot from the Ts measurement of compound **4** in Agarose gel including the delays with the respective normalized integrals and the obtained Ts value.

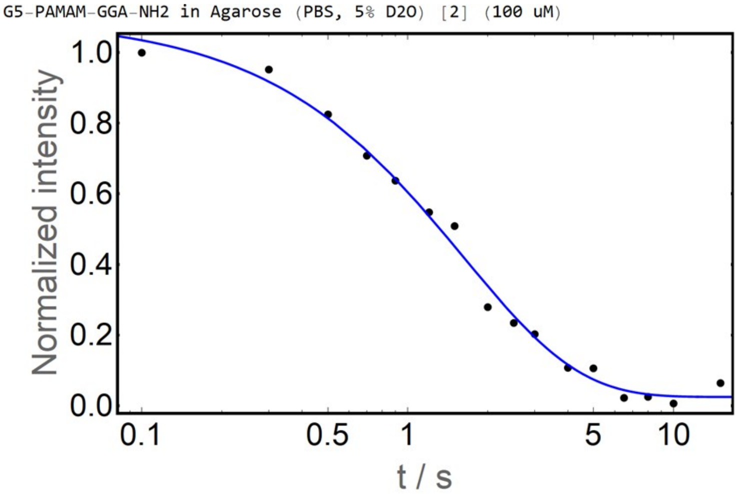

Data	
t	Integrals
0.100	1.000
0.300	0.952
0.500	0.825
0.700	0.708
0.900	0.637
1.200	0.547
1.500	0.509
2.000	0.279
2.500	0.235
3.000	0.202
4.000	0.108
5.000	0.106
6.500	0.023
8.000	0.025
10.000	0.006
15.000	0.064

$y = M1 \times [1 - \text{Exp}(-t/\tau 1)] + c1$

	Estimate	Standard Error	t-Statistic	P-Value
M1	−1.07396	0.0271071	−39.6191	6.06414×10^{-15}
τ1	1.62341	0.0996761	16.2868	4.98073×10^{-10}
c1	1.0988	0.0264256	41.5808	3.25026×10^{-15}

Figure S47: Plot from the Ts measurement of compound **4** in Agarose gel including the delays with the respective normalized integrals and the obtained Ts value.

NMR spectra of (2) and (4)

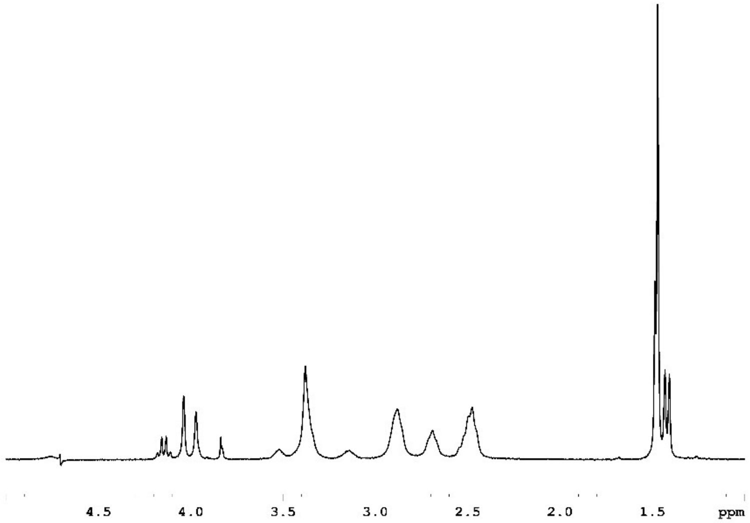

Figure S48: Proton spectrum of a 100 µM solution of **2** in D_2O with presaturation on the solvent signal at a magnetic field of 7.05 T. Two scans were run in this experiment with an inter-scan delay of *d1*=5 s. The 90° pulse was *p1*=16 µs.

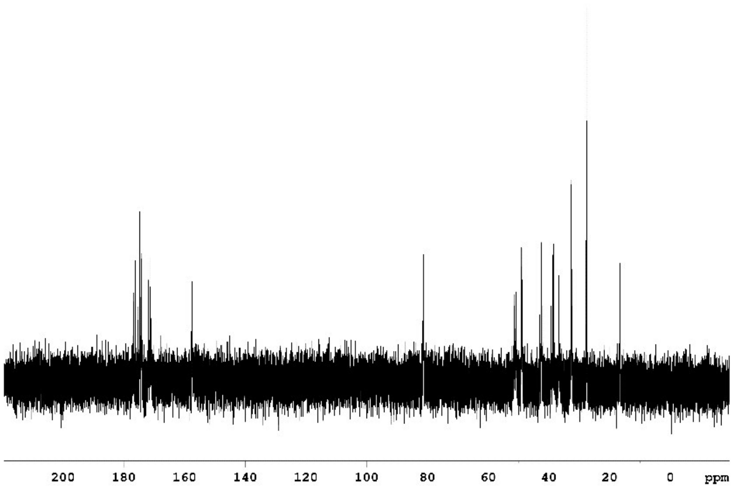

Figure S49: ^{13}C spectrum of a concentrated solution of **2** in D_2O at a magnetic field of 7.05 T.. 8192 scans were run in this experiment with an inter-scan delay of *d1*=2 s. The 90° pulse was *p1*=2.5 µs with a 30° flip angle.

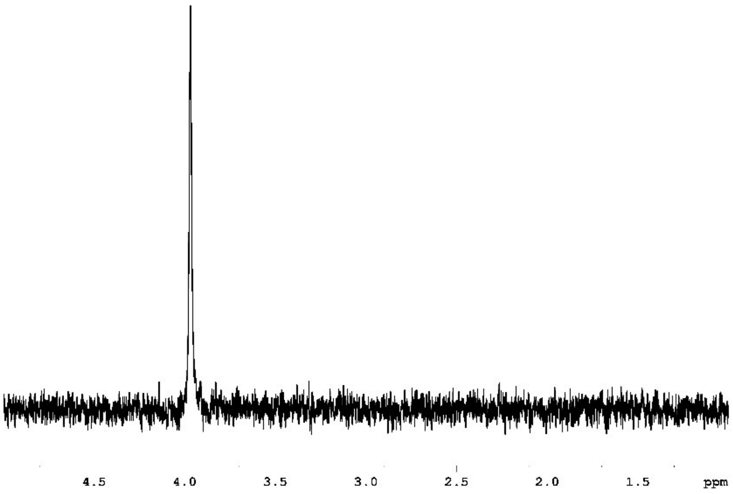

Figure S50: Singlet state NMR spectrum of a 100 µM solution of **2** in D2O at a magnetic field of 7.05 T. Four scans were run in this experiment with an inter-scan delay of *d1*=15 s. The 90° pulse was *p1*=16 µs and the SLIC pulse length was *d21*=80 ms at 17 Hz nutation frequency.

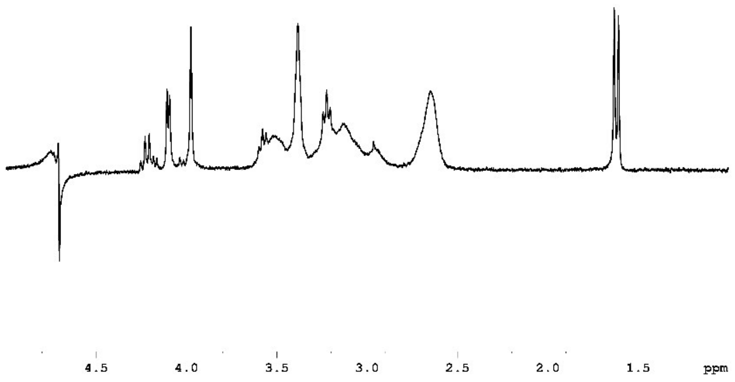

Figure S51: Proton spectrum of a 100 µM solution of **4** in D_2O with presaturation on the solvent signal at a magnetic field of 7.05 T. Two scans were run in this experiment with an inter-scan delay of *d1*=5 s. The 90° pulse was *p1*=15 µs.

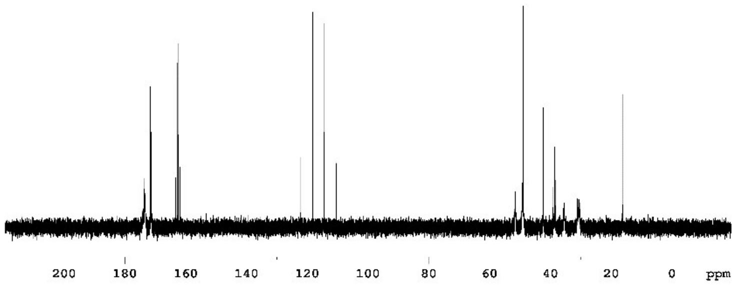

Figure S52: ^{13}C spectrum of a concentrated solution of **4** in D_2O at a magnetic field of 7.05 T.. 4096 scans were run in this experiment with an inter-scan delay of *d1*=2 s. The 30° pulse was *p1*=2.5 µs.

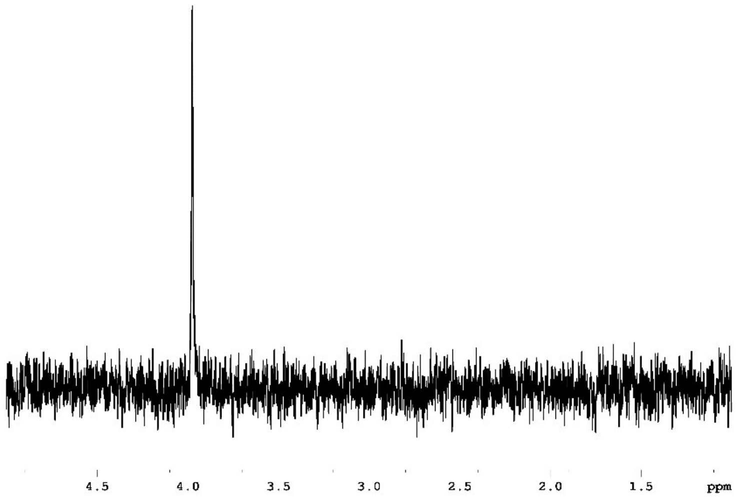

Figure S53: Singlet state NMR spectrum of a 100 µM solution of **4** in D2O at a magnetic field of 7.05 T.. Eight scans were run in this experiment with a inter-scan delay of *d1*=30 s. The 90° pulse was *p1*=15 µs and the SLIC pulse length was *d21*=310 ms at 17 Hz nutation frequency.

IR spectra of the starting material as well as of (2) and (4)

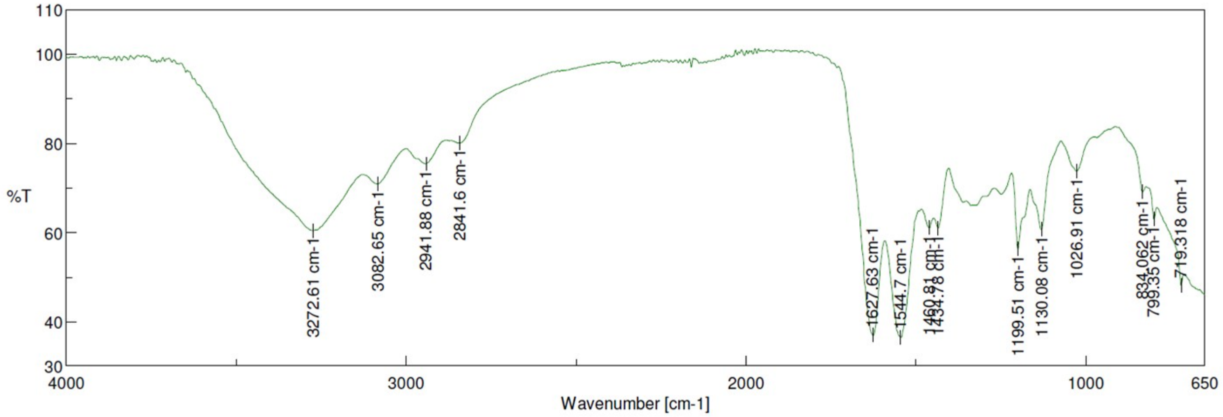

Figure S54: IR spectrum of the starting material G5-PAMAM-NH_2.

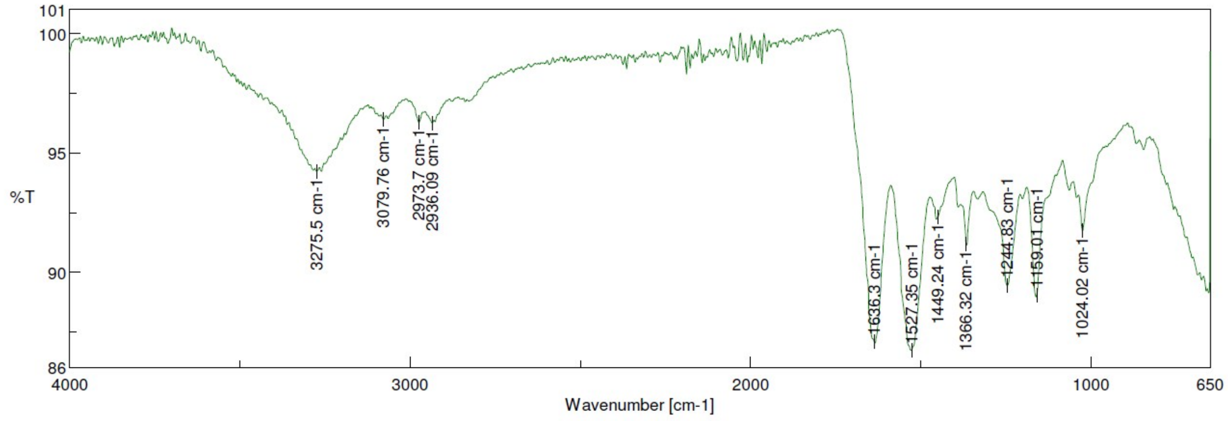

Figure S55: IR spectrum of **2**.

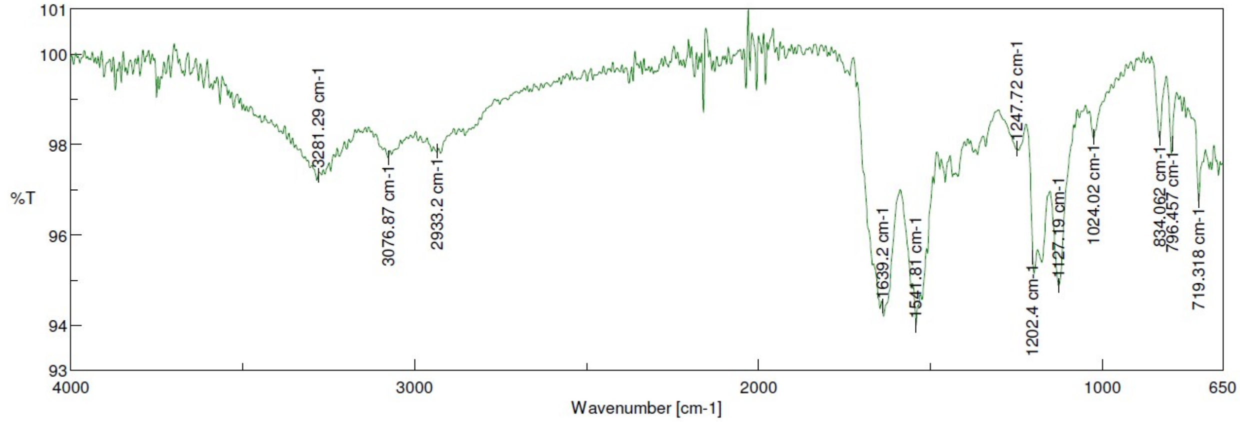

Figure S56: IR spectrum of **4**.

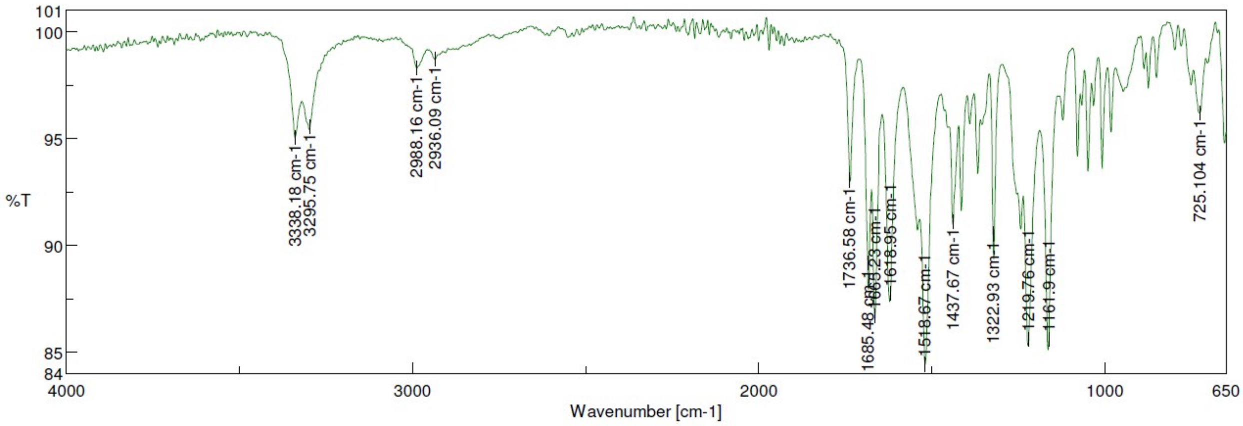

Figure S57: IR spectrum of **1**.

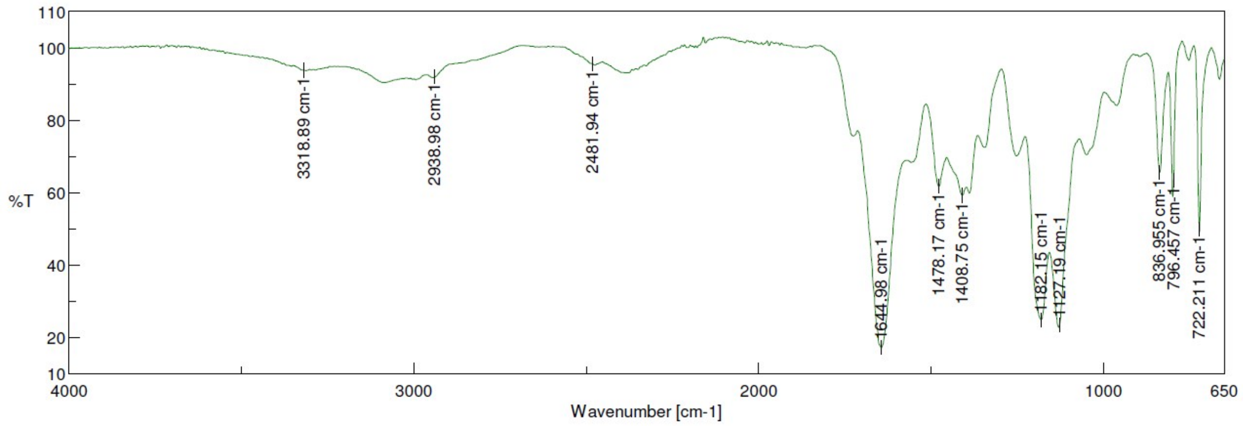

Figure S58: IR spectrum of **3**.

References

1 S. J. DeVience, R. Walsworth and M. S. Rosen, *Phys. Rev. Lett.* **2013**, *111*, 173002.
